# Polyesterase activity and thermostability of carboxylesterases from *Thermoleophilum album* YS‐3

**DOI:** 10.1111/febs.70478

**Published:** 2026-03-09

**Authors:** Tatyana N. Chernikova, Anna N. Khusnutdinova, Hairong Ma, Manuel Ferrer, Olga V. Golyshina, Alexander F. Yakunin, Peter N. Golyshin

**Affiliations:** ^1^ Centre for Environmental Biotechnology, School of Environmental and Natural Sciences Bangor University UK; ^2^ Instituto de Catalisis y Petroleoquimica (ICP), CSIC Madrid Spain

**Keywords:** carboxylesterase, polycaprolactone, polyester degradation, polyesters, polyethylene terephthalate, polylactic acid, *Thermoleophilum album*, thermostability

## Abstract

Recently, enzymatic depolymerisation of synthetic polyesters has emerged as an attractive complement to current plastic recycling methods. However, the arsenal of robust enzymes available for these applications remains limited. Here, we identified and biochemically characterised three novel carboxylesterases (TA21, TA26 and TA29) with polyesterase activity from the genome of the thermophilic, obligately alkane‐degrading bacterium *Thermoleophilum album* YS‐3 (ATCC 35264). The purified proteins hydrolysed model *para*‐nitrophenyl monoesters, favouring short‐chain substrates (C2–C6), with TA21 showing the highest carboxylesterase activity. All three enzymes displayed maximal activity at 55–60 °C, with 20–25% activity remaining at 75 °C. Notably, they also retained substantial activity at moderate temperatures (15 °C), which is uncommon for thermophilic enzymes. In agarose‐plate screens with emulsified polyesters, the enzymes hydrolysed amorphous poly(ethylene terephthalate) (aPET), bis(benzoyloxyethyl) terephthalate (3PET), polylactic acid (PLA) and polycaprolactone (PCL). HPLC analysis identified terephthalic acid (TA) and mono(2‐hydroxyethyl) terephthalate (MHET) as the major degradation products of 3PET and aPET, with TA21 efficiently converting MHET to TA. TA21 was active on PLA and PCL, hydrolysing them to lactic acid and 6‐hydroxyhexanoic acid, respectively, and preferred 3PET and PLA over aPET and PCL (0.25–1.5 mm products formed after overnight incubation). Structural analysis revealed the presence of a medium‐sized lid domain in all three enzymes. In TA21, this domain contributes two aromatic residues and an arginine for coordinating MHET in the active site, which may account for its higher MHET‐degrading activity. These findings introduce novel thermophilic polyesterases that may serve as promising candidates for further optimisation and protein engineering research on enzymatic depolymerisation of synthetic polyesters.

## Graphical abstract

Three novel α/β‐hydrolases from thermophilic bacterium *Thermoleophilum album* display carboxylesterase and polyesterase activity. These enzymes hydrolyse PET, PLA and PCL both at high and moderate temperatures. TA21 shows superior activity, efficiently converting MHET to terephthalic acid. Structural features underlying substrate binding highlight their potential for enzymatic polyester depolymerisation.

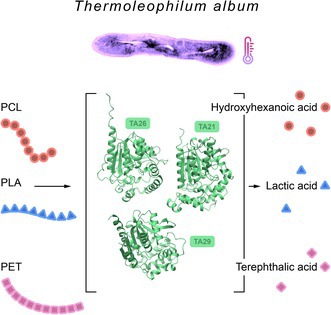

**Abbreviations**
± SEM: standard error of the mean
3‐PET: bis(benzoyloxyethyl) terephthalate
BA: benzoic acid
BHET: bis(2‐hydroxyethyl) terephthalate
DSF: differential scanning fluorimetry
HEB: hydroxyethyl benzoate
*Is*PETase: *Ideonella sakaiensis* PETase
LCC: leaf‐branch compost cutinase
MHET: mono(2‐hydroxyethyl) terephthalate
PBAT: poly(butylene adipate‐co‐terephthalate)
PBS: poly(butylene succinate)
PBT: polybutylene terephthalate
PCL: polycaprolactone
PEA: poly(ethylene adipate)
PEF: poly(ethylene furanoate)
PEN: poly(ethylene naphthalate)
PET: polyethylene terephthalate
PGA: polyglycolic acid
PHB: polyhydroxybutyrate
PLA: polylactic acid
*p*NP: *para*‐nitrophenyl
PTT: poly(trimethylene terephthalate)
TA: terephthalic acid

## Introduction

Plastics are currently the most widely consumed synthetic products, owing to their remarkable chemical diversity, durability, elasticity, lightness and other advantageous properties, as well as low production costs [1]. Over the past decade, plastic production has risen by 35% and is projected to reach 700 million metric tons (Mt) in 2030 (roughly 80 kg of plastics per person), while the current polymer economy remains largely linear [2, 3, 4]. Of the nearly nine billion tons of plastics produced since the 1950s, about 80% has been landfilled, 10–12% incinerated and less than 10% recycled. The high chemical and structural stability that makes plastics valuable in many applications also hinders their conversion into new products and, more critically, their biodegradation in the environment and landfills [3, 5, 6, 7]. Furthermore, the environmental degradation of plastic waste is hindered by the presence of various antioxidants and stabilisers, which are added to extend the lifespan of plastics [1, 8]. Plastics persist in the environment, with natural removal occurring over timescales ranging from decades to centuries [9]. However, inadequate end‐of‐life management of plastics has become a global environmental threat, disrupting ecosystems worldwide and posing serious health risks [10]. This also leads to the loss of valuable organic materials and the depletion of nonrenewable petroleum feedstocks needed to produce new virgin plastics. Efficient plastic recycling is therefore crucial for reducing energy consumption, CO_2_ emissions and global warming, as well as for the recovery of organic chemicals and conservation of fossil feedstocks.

Currently, plastics are recycled primarily through thermo‐mechanical and chemical methods, which often yield downgraded materials and involve high energy use and toxic reagents [2, 11, 12]. In this context, enzymatic plastic recycling is emerging as a complementary approach, which is based on enzymatic depolymerisation of synthetic polymers to original monomers and can be used in combination with mechanical or chemical plastics pretreatment [2, 3, 4, 13, 14, 15]. Hydrolysable commodity plastics, such as polyethylene terephthalate (PET), represent a large group of polyester plastics, which accounted for over 10% of the total plastic production in 2023 [4]. Other common synthetic polyesters include poly(lactic acid) (PLA), polycaprolactone (PCL), poly(butylene succinate) (PBS), poly(ethylene adipate) (PEA), polyglycolic acid (PGA), poly(butylene adipate terephthalate) (PBAT), polybutylene terephthalate (PBT), poly(trimethylene terephthalate) (PTT), and poly(ethylene naphthalate) (PEN). Natural polyesters are also known, such as cutin (a structural component of the plant cuticle), polyhydroxybutyrate (PHB, bacterial and archaeal storage polyester), and shellac (secreted by some insects). The first reports on enzymatic degradation of PET and other polyesters by purified microbial lipases and hydrolases were published 50 years ago and later [16, 17, 18]. To date, over 300 plastics‐active enzymes have been catalogued in the PAZy (Plastics‐Active Enzymes) and PlasticDB databases [19, 20]. In 2012, a cutinase‐like enzyme (LCC) with PET‐degrading activity was isolated from the leaf‐branch compost metagenome, which remains one of the most active PETases known to date [21, 22]. Subsequently, a two‐enzyme system comprising two carboxylesterases (*Is*PETase and *Is*MHETase) from the mesophilic bacterium *Ideonella* (*Piscinibacter*) *sakaiensis* was characterised and reported to degrade PET to a monoester intermediate (MHET, via *Is*PETase) and then to terephthalic acid and ethylene glycol (by *Is*MHETase) [23]. Currently, the majority of known natural polyester‐degrading enzymes are PET hydrolases (PETases, over 200 proteins), while smaller numbers of polyesterases have been reported for PLA (41 proteins), PBAT (21 proteins), PHB (17 proteins), as well as PCL, PBS, and poly(ethylene furanoate) (PEF) [19, 24, 25, 26, 27]. Crystal structures have been determined for several enzymes, including several bacterial carboxylesterases (from *Clostridium hathewayi*, *C. botulinum, Rhodopseudomonas palustris*, and *I. sakaiensis*) and structurally‐related cutinases (from *Thermobifida alba*, *T. fusca*, and the leaf‐branch compost) [22, 28, 29, 30, 31, 32]. Later, the wild‐type LCC cutinase was engineered for enhanced PET depolymerisation and successfully applied for the transformation of PET waste (plastic bottles and polyester textiles) to PET monomers, which were then repolymerised into virgin PET and used to produce new PET‐based products [33]. Recently, several new natural PET‐degrading hydrolases and engineered variants of known PETases (LCC and PES‐H1) have been reported with activity and stability comparable to or exceeding those of the wild‐type LCC [34, 35, 36].

Various biochemical studies have demonstrated that enzymatic polyester depolymerisation proceeds more efficiently near the glass transition temperature of amorphous polyesters (65–80 °C for PET), due to increased polyester chain mobility [27, 33, 37, 38]. Therefore, thermophilic and thermostable polyester depolymerising enzymes are preferred over mesophilic biocatalysts [39]. To prevent the rapid spontaneous crystallisation of amorphous PET at ≥ 75 °C, polyesterases with optimal temperatures at 72–74 °C are particularly desirable for PET recycling applications [34]. Consequently, sequence‐ and activity‐based mining of novel thermophilic polyesterases, together with protein engineering efforts to improve the thermostability of mesophilic PETases, have been carried out recently and are still ongoing [27, 34, 35, 40, 41]. For example, protein engineering of the mesophilic *Is*PETase has yielded several improved variants (ThermoPETase, HotPETase, FAST‐PETase, DuraPETase), capable of degrading postconsumer PET at temperatures between 30 °C and 72 °C [42, 43, 44, 45]. However, these studies and sequence‐based mining yield polyesterase variants confined to a relatively narrow sequence space (with high similarity to known PETases), highlighting the need to explore and discover new classes of polyester‐degrading enzymes as potential leads for protein and metabolic engineering [46]. This is evidenced by the discovery of several thermophilic cutinases with PETase activity in the genomes of different *Thermobifida* species, which are commonly found in decaying plant materials and composts [2, 16, 37, 47, 48, 49].

In this study, we explored the uncharacterised α/β‐hydrolases from the thermophilic alkane‐degrading bacterium *Thermoleophilum album* YS‐3 for the presence of thermostable polyester‐degrading enzymes. *T. album* is obligate for thermophily and alkane substrates and grows on aliphatic hydrocarbons (C_9_–C_26_) at optimal temperatures 60–70 °C [50, 51, 52]. This bacterium is present in diverse terrestrial and aquatic environments, including hot springs, but is also abundant in communities associated with agricultural composts, suggesting a potential role in the biodegradation of natural polymers, including the plant polyester cutin [53]. Here, we cloned eight α/β hydrolases predicted in *T. album* for recombinant expression in *E. coli*. Three clones (TA21, TA26, and TA29) produced soluble proteins, which were biochemically characterised using both monoester and polyester substrates. All three enzymes exhibited a preference to short‐chain substrates (C2‐C6) and optimal activity at pH 7.5–9.0 and elevated temperatures (45–65 °C). Agarose screens and HPLC assays revealed the hydrolytic activity against amorphous PET (aPET), the short‐chain model PET‐like substrate bis(benzoyloxyethyl) terephthalate (3PET), polylactic acid (PLA), and polycaprolactone (PCL) in all three proteins. At elevated temperatures (60–70 °C), the *T. album* polyesterases degraded aPET and PLA with the rates comparable to those of reference PETases (FastPETase and HotPETase). Structural analysis of the *T. album* enzymes showed the presence of lid domains in these proteins, which contribute two aromatic residues and one arginine for coordinating MHET in the active site of TA21. Thus, the α/β hydrolases from *T. album* represent promising polyesterases for potential applications in polyester recycling and upcycling.

## Results and Discussion

### Expression and purification of selected α/β hydrolases from *T. album*

Although the *Thermoleophilum* genus was first described nearly 40 years ago, only two enzymes from *T. album* have been characterised to date: a manganese‐containing catalase and malate dehydrogenase [54, 55]. These enzymes retained significant activity at assay temperatures from 25 °C to 80 °C, with optimal activity at 60 °C.

The complete genome of *T. album* YS‐3 (ATCC 35264) was sequenced in 2020 in our lab (GenBank CP066171), revealing over 60 genes encoding putative hydrolases with 37 predicted α/β hydrolases and metallo‐hydrolases, which may represent potential enzymes with carboxylesterase activity. A standard BLAST search (NCBI) using the sequences of known reference polyesterases and cutinases (*Is*PETase, LCC, TfCut1) revealed no close homologues or proteins with significant sequence similarity in the *T. album* genome. As a first step in identifying novel polyesterases from *T. album*, we cloned eight predicted α/β hydrolases for recombinant expression in *E. coli* (Tables S1 and S2).

According to the Arpigny–Jaeger classification [56], the selected α/β hydrolases from *T. album* belong to carboxylesterase families I (TA3, TA8), II (TA4), IV (TA21), and V (TA9, TA20, TA26, and TA29) (Table S1). In the sequences of TA3, TA4, TA8, and TA9, potential signal peptides were predicted by the SignalP 6.0 server and were removed prior to cloning for expression in *E. coli*. Following expression and affinity purification, three recombinant *E. coli* clones (TA21, TA26, and TA29) yielded soluble proteins with the expected molecular masses (38, 35, and 32 kDa, respectively) (t). As expected for predicted α/β hydrolases, the amino acid sequences of these proteins revealed the classical Ser‐His‐Asp catalytic triad characteristic for Ser‐hydrolases (Figs S1 and S2). The TA21 sequence shared 46–48% sequence identity to the alkaline carboxylesterase Est8 from an oil‐contaminated soil metagenome and LipN from *Mycobacterium tuberculosis*, as well as comparable sequence similarity with several thermophilic carboxylesterases, including EST2 from *Alicyclobacillus acidocaldarius*, AFEST from *Archaeoglobus fulgidus*, and PestE from *Pyrobaculum calidifontis* [57, 58, 59, 60, 61] (Fig. S1). TA26 shows 32% sequence identity to the phospholipase PlaF from *Pseudomonas aeruginosa*, whereas the TA29 sequence exhibits 33% identity with a metagenomic esterase from Amazonian soil [62, 63] (Fig. S4). Both TA26 and TA29 belong to the carboxylesterase family V, but their sequences share low similarity to each other, with only 29% identity.

Phylogenetic analysis of TA21 and other biochemically characterised family IV carboxylesterases revealed that its relationship to a large cluster of mesophilic and thermophilic bacterial and fungal enzymes, including solvent tolerant Est8 from *Parvibaculum* and rPPE from *Pseudomonas putida* [57, 64] (Fig. S3). In contrast, the family V enzyme TA26 was found to be associated with the C‐C bond hydrolase BphD from *Burkholderia xenovorans* and the CumD hydrolase from the cumene degradation pathway in *Pseudomonas fluorescens*, whereas TA29 clustered with the RdmC methylesterase from *Streptomyces purpurascens* [65, 66, 67] (Fig. S4).

### Carboxylesterase activity of purified *T. album* α/β hydrolases

**Fig. 1:**
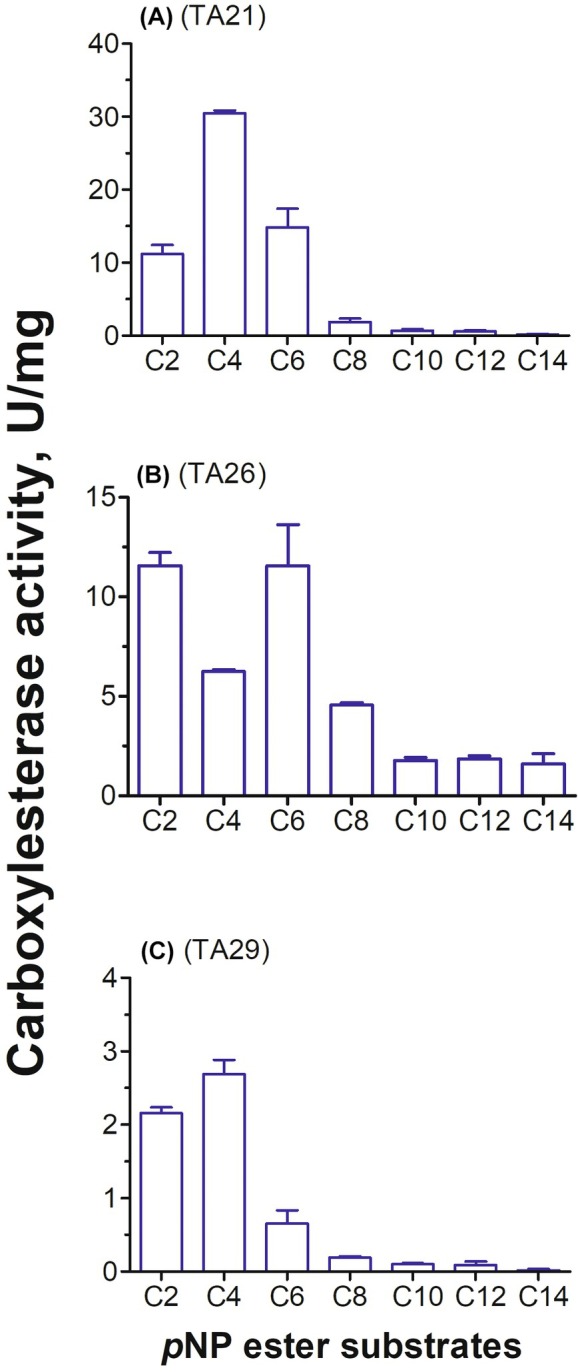
Carboxylesterase activity of purified α/β hydrolases from *T. album* against chromogenic *p*‐nitrophenyl (*p*NP) esters with varying acyl chain lengths: (A), TA21; (B), TA26; (C), TA29. Carboxylesterase activity was measured using 1 mm substrates: *p*NP‐acetate (C2), *p*NP‐butyrate (C4), *p*NP‐hexanoate (C6), *p*NP‐octanoate (C8), *p*NP‐dodecanoate (C12), *p*NP‐tetradecanoate (C14). Reaction mixtures (0.2 mL) contained 1 μg of purified protein and 50 mm Tris–HCl buffer (pH 8.0, 20 min incubation at 30 °C). Data represent means ± standard error of the mean (SEM) from at least three independent determinations.

To demonstrate the presence of carboxylesterase activity in TA21, TA26 and TA29, the purified proteins were assayed with a panel of chromogenic *p*‐nitrophenyl (*p*NP) monoesters with varying acyl chain lengths. For an initial assessment, these reactions were conducted at 30 °C, a commonly used assay temperature for enzymes derived from environmental microorganisms. As shown in Fig. 1, all proteins displayed maximal carboxylesterase activity towards *p*NP‐monoesters with short acyl chains (C2–C6). With these substrates, TA21 was the most active carboxylesterase with specific activities up to 30 U·mg^−1^, whereas TA26 exhibited higher activity against long acyl chain substrates (C10–C14) than the other *T. album* enzymes tested (Fig. 1). However, these enzymes revealed no hydrolytic activity toward natural oils, including coconut, palm and olive oils. Purified *T. album* carboxylesterases showed saturation kinetics and high catalytic rates toward short‐ and medium‐chain *p*NP‐monoesters (C2 to C8) (Table 1). Both TA26 and TA29 displayed higher substrate affinity (lower *K*_m_) than TA21, and all three enzymes exhibited greater affinity to medium‐chain *p*NP‐monoesters (C6 and C8). Among the tested enzymes, TA21 and TA29 showed the highest catalytic efficiency (*k*_cat_/*K*_m_) with *p*NP‐hexanoate, whereas the greatest efficiency of TA26 was observed with *p*NP‐acetate and *p*NP‐butyrate (Table 1). Therefore, *p*NP‐butyrate and *p*NP‐hexanoate were selected as monoester substrates for further biochemical characterisation of the *T. album* carboxylesterases. Overall, the kinetic parameters of the tested *T. album* enzymes with *p*NP‐monoesters were comparable to those reported for other microbial carboxylesterases [31, 57, 58, 63].

**Table 1:** Kinetic parameters of purified TA21, TA26 and TA29 with model esterase substrates.^a^

Proteins	Variable substrates	*K*_m_, mm	*k*_cat_, s^−1^	*k*_cat_/*K*_m_, m ^−1^·s^−1^
TA21	*p*NP‐acetate (C2)	5.4 ± 0.5	54.3 ± 3.1	0.95 ± 0.1 × 10^4^
	*p*NP‐butyrate (C4)	2.2 ± 0.4	21.9 ± 4.8	1.0 ± 0.2 × 10^4^
	*p*NP‐hexanoate (C6)	1.3 ± 0.2	59.9 ± 3.4	0.5 ± 0.1 × 10^5^
	*p*NP‐octanoate (C8)	0.9 ± 0.2	18.8 ± 0.3	2.3 ± 0.6 × 10^4^
TA26	*p*NP‐acetate (C2)	0.8 ± 0.1	112.6 ± 4.3	1.5 ± 0.1 × 10^5^
	*p*NP‐butyrate (C4)	2.9 ± 0.3	337.4 ± 15.3	1.1 ± 0.1 × 10^5^
	*p*NP‐hexanoate (C6)	1.5 ± 0.4	11.6 ± 1.2	0.8 ± 0.1 × 10^4^
	*p*NP‐octanoate (C8)	0.4 ± 0.04	21.9 ± 0.6	0.5 ± 0.01 × 10^5^
TA29	*p*NP‐acetate (C2)	1.9 ± 0.2	9.6 ± 0.4	5.1 ± 0.4 × 10^3^
	*p*NP‐butyrate (C4)	1.1 ± 0.1	29.6 ± 0.5	2.7 ± 0.1 × 10^4^
	*p*NP‐hexanoate (C6)	0.4 ± 0.1	36.4 ± 2.0	0.9 ± 0.2 × 10^5^
	*p*NP‐octanoate (C8)	0.4 ± 0.2	33.0 ± 3.8	0.9 ± 0.3 × 10^5^

^a^ The values represent mean ± SEM from at least three experiments.

### Optimal reaction conditions and stability of TA21, TA26, and TA29

**Fig. 2:**
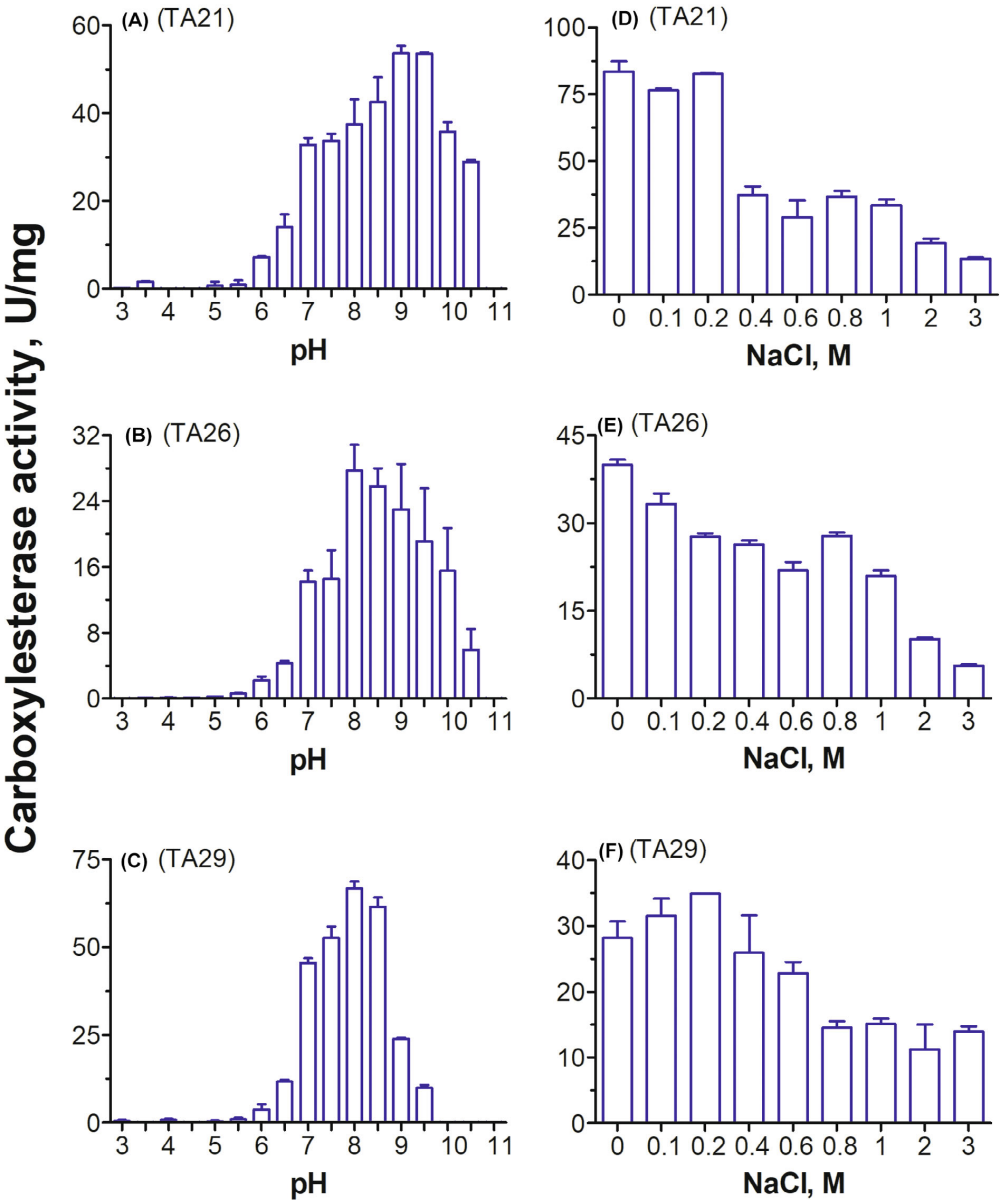
Carboxylesterase activity of purified *T. album* α/β hydrolases: the effect of pH and NaCl. (A, B, C), effect of varying pH; (D, E, F), effect of increasing NaCl concentrations. TA21 (*p*NP‐hexanoate, C_6_); (B), TA26 (*p*NP‐hexanoate, C_6_); (C), TA29 (*p*NP‐butyrate, C_4_). Reaction mixtures (0.2 mL) contained 50 mm Britton‐Robinson buffer with the indicated pH (A, B, C) or 50 mm Tris–HCl buffer (pH 9.0 for TA21 or pH 8.0 for TA26 and TA29), 1 mm substrate (*p*NP‐hexanoate for TA21 and TA26, *p*NP‐butyrate for TA29) and 1 μg of purified protein (20 min incubation at 30 °C). Results are means ± SEM from at least three independent determinations.

With *p*NP‐monoesters as substrates, the purified *T. album* carboxylesterases exhibited significant hydrolytic activity across a broad pH range, with maximal activity at pH 7.0–10.5 for TA21 and TA26 and within a narrower range (pH 7.0–9.0) for TA29 (Fig. 2A–C). A similar pH range has been reported for biochemically characterised carboxylesterases from both mesophilic and thermophilic bacteria [24, 31, 40, 57, 68, 69, 70]. Several studies with purified carboxylesterases from various metagenomes and microorganisms have demonstrated significant stimulation of activity by moderate or high concentrations of NaCl [31, 69, 70, 71]. Both TA21 and TA29 tolerated well low NaCl concentrations (up to 0.2–0.4 m) (Fig. 2D,F), whereas TA26 showed a steady decline in carboxylesterase activity with increasing NaCl concentrations up to 3 m (Fig. 2E). Nevertheless, all three enzymes retained significant activity at high NaCl levels (1–3 m), corresponding to 20–40% of their activity without NaCl (Fig. 2D–F). Although several microbial carboxylesterases have been reported to be stimulated by low concentrations of the non‐ionic detergent Tween 20 [24, 69, 72], the carboxylesterase activity of the analysed *T. album* enzymes gradually decreased in the presence of Tween 20 with near complete inactivation observed at 3.2% detergent (Fig. S5).

**Fig. 3:**
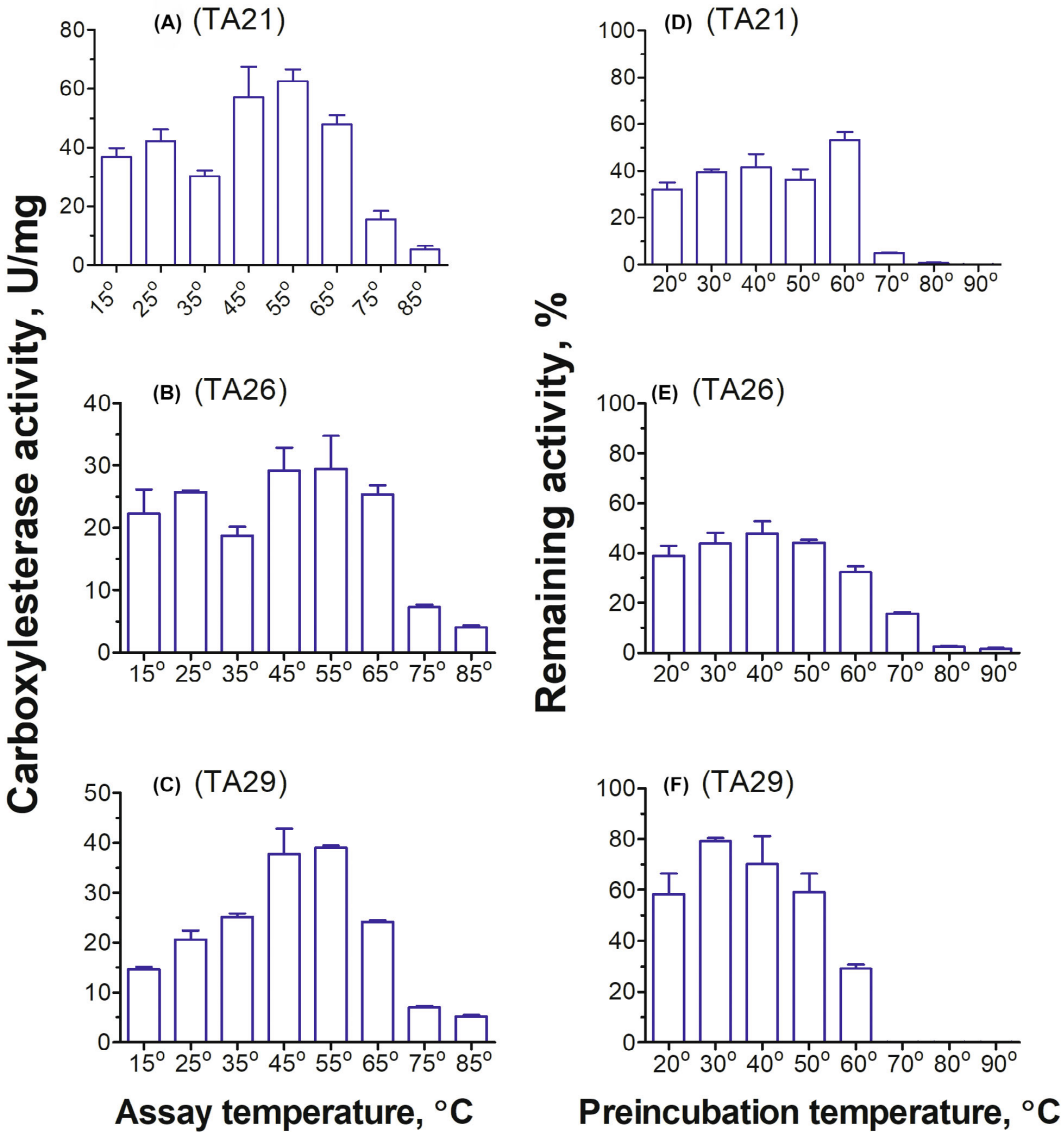
Effect of temperature on the carboxylesterase activity and thermostability of purified *T. album* α/β hydrolases. (A, B, C), Carboxylesterase activity of TA21 (A), TA26 (B), and TA29 (C) at different temperatures; (D, E, F), Residual activity of TA21 (D), TA26 (E), and TA29 (F) after 1 h preincubation at the indicated temperatures as specified in the Section “Materials and Methods”. Reaction mixtures (0.2 mL) contained 50 mm Tris–HCl buffer (pH 9.0 for TA21 or pH 8.0 for TA26 and TA29), 1 mm substrate (*p*NP‐hexanoate for TA21 and TA26; *p*NP‐butyrate for TA29), and purified proteins: 0.05 μg (A, B, C; 20 min incubation at indicated temperatures) or 1 μg (D, E, F; 20 min incubation at 30 °C). Residual activities were calculated as percentages of the initial activity (100%) of freshly thawed proteins before preincubation at indicated temperatures (TA21: 72.0 ± 4.3 U·mg^−1^; TA26: 71.2 ± 9.1 U·mg^−1^; TA29: 61.0 ± 1.3 U·mg^−1^). Data represent means ± SEM from at least three independent determinations.

As expected for enzymes derived from a thermophilic bacterium, the three *T. album* carboxylesterases exhibited the highest activity at 55 °C and retained 10–25% of the maximal activity at 75–85 °C (Fig. 3A–C). Surprisingly, these enzymes also tolerated low assay temperatures with 40–70% of the peak activity at 15 °C, suggesting that they can function effectively at both low and high extreme temperatures. The thermostability of purified *T. album* carboxylesterases was evaluated using two approaches: thermal denaturation analysis by differential scanning fluorimetry (Fig. S6) and determination of residual enzyme activity after 1 h preincubation at various temperatures (Fig. 3D–F). Differential scanning fluorimetry (DSF) monitors protein denaturation in the presence of the fluorescent dye SYPRO Orange as a function of temperature, allowing determination of the protein melting temperature (*T*_m_) (see Materials and Methods for experimental details). For the purified TA29, the melting temperature could not be determined due to a high initial fluorescence background at low temperatures, suggesting the presence of substantial amounts of misfolded protein (Fig. S6). DSF analyses with TA21 and TA26 yielded typical thermodenaturation profiles, with melting temperatures (*T*_m_) of 74.9 °C and 68.6 °C, respectively (Fig. S6). Although these *T*_m_ values are slightly lower than those of thermophilic reference PETases (84.7 °C for LCC and 82.5 °C for HotPETase [33, 43]), they are nevertheless significantly higher than the reported *T*_m_ of the mesophilic *Is*PETase (46.8 °C) [73]. Analysis of the residual carboxylesterase activity (measured at 30 °C) of purified *T. album* enzymes after 1 h of preincubation at various temperatures revealed no detectable activity in TA29 after preincubation at 70 °C, whereas TA21 and TA26 retained 5–15% of their activity (Fig. 3B). These observations are consistent with the DSF analysis results (Fig. S6), as such a degree of thermostability is characteristic of thermophilic enzymes, whereas most mesophilic proteins lose their activity after preincubation at 40–50 °C [40, 69]. As shown in Fig. 3B, TA21 and TA26 also lost a substantial portion of their initial activity (up to 60%) after preincubation at lower temperatures (20–30 °C), suggesting that, similar to the *Pyrobaculum calidifontis* carboxylesterase Est [68], they may be unstable at low protein concentrations. Overall, consistent with their activity temperature profiles and DSF analysis, the analysed *T. album* carboxylesterases display notable thermostability and high‐temperature tolerance while also maintaining significant activity at low and moderate temperatures.

### Analysis of polyesterase activity of purified *T. album* carboxylesterases

**Fig. 4:**
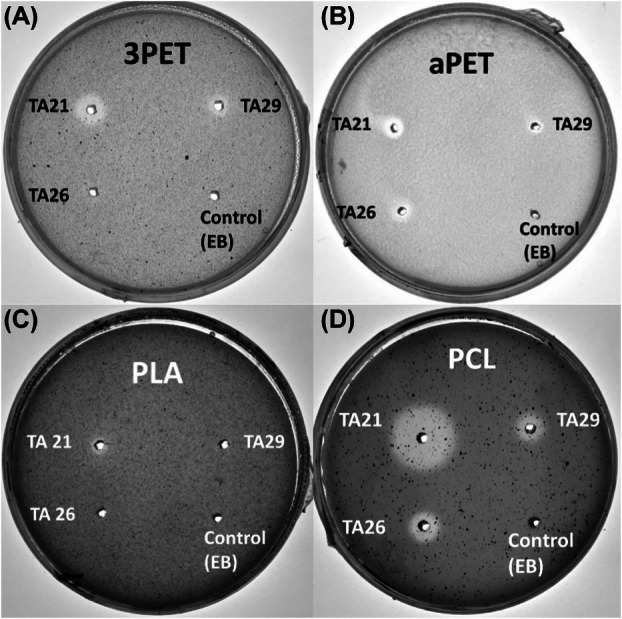
Agarose‐clearing screening of purified *T. album* α/β hydrolases for hydrolytic activity against emulsified polyester substrates. (A), bis(benzoyloxyethyl) terephthalate (3PET); (B), amorphous PET (aPET); (C), polylactic acid (PLA); (D), polycaprolactone (PCL). Agarose plates (2% w/v) containing 0.125% of the indicated emulsified polyester were prepared in 50 mm Tris–HCl buffer (pH 8.0) as described in Materials and Methods. Agarose wells were loaded with 10 μg of purified enzymes and incubated at 37 °C for 48 h, followed by 24 h at 60 °C. The protein elution buffer (EB) used during affinity purification served as the negative control.

As noted above, the selected *T. album* α/β hydrolases belong to the carboxylesterase families IV and V, which encompass most of the known PET‐degrading enzymes including LCC and *Is*PETase [21, 23]. The presence of polyesterase activity in purified *T. album* carboxylesterases was initially evaluated using agarose‐based screens with several emulsified polyesters, including bis(benzoyloxyethyl) terephthalate (3PET), amorphous PET (aPET), polylactic acid (PLA), and polycaprolactone (PCL). The purified *T. album* enzymes were loaded into wells cut into solidified agarose containing emulsified polyesters, and the plates were incubated at 37 °C (for 48 h) and then at 60 °C (for 24 h). As shown in Fig. 4, TA21 exhibited polyesterase activity (the formation of clear zones) against all tested substrates, TA26 hydrolysed aPET and PCL, whereas TA29 showed activity toward 3PET, aPET, and PCL. Based on the size of the clear zones around the wells, TA21 was the most active polyesterase among the three tested *T. album* enzymes, while aPET and PLA (poly(D,L‐lactide), M_w_ range 10 000–18 000) were more resistant to degradation than 3PET and PCL (Fig. 4). Although 3PET is commonly used as a model PET substrate [47, 74], these screens revealed that 3PET‐degrading activity does not always correlate with hydrolytic activity against aPET (Fig. 4).

**Fig. 5:**
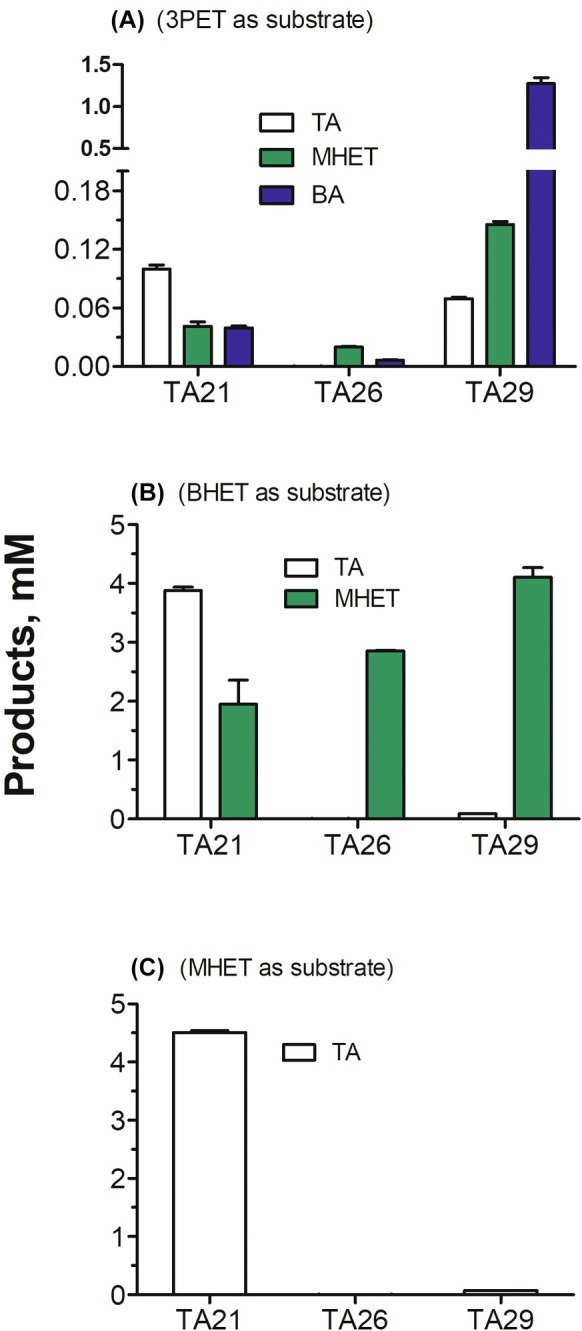
Hydrolytic activity of *T. album* α/β hydrolases against 3PET and its degradation intermediates: HPLC analysis of reaction products. (A), 3PET as substrate (0.125%); (B), BHET as substrate (5 mm); (C), MHET as substrate (5 mm). The reaction mixtures (0.2 mL) contained 50 mm Tris–HCl buffer (pH 9.0 for TA21 or pH 8.0 for TA26 and TA29), the indicated substrate, and 10 μg of purified protein (incubation at 30 °C for 16 h). Results are means ± SEM from at least three independent determinations.

Next, the polyesterase activity of *T. album* carboxylesterases was evaluated by HPLC analysis of soluble reaction products after incubating the purified enzymes with emulsified 3PET at 30 °C for 16 h (Fig. 5). As reported in previous studies [75, 76], hydrolysis of 3PET, which contains four ester bonds, is expected to generate several aromatic products: bis(2‐hydroxyethyl) terephthalate (BHET; a diester), mono(2‐hydroxyethyl) terephthalate (MHET; a monoester), benzoic acid (BA), hydroxyethylbenzoate (HEB) and terephthalic acid (TA) (Fig. S7). HPLC analysis of soluble reaction products of 3PET degradation revealed that the purified *T. album* polyesterases produced significant amounts of TA, MHET and BA (Fig. 5A). These assays detected no measurable amounts of the 3PET degradation intermediates BHET and HEB, indicating that they were completely degraded by the *T. album* enzymes. For TA21, TA was the predominant product with lower levels of MHET and BA, whereas TA29 mainly produced BA, suggesting a preference for cleavage at the 3PET terminal ester bonds (exo‐cleavage) (Fig. 5A). Furthermore, our results indicate that HPLC‐based polyesterase assays are more sensitive than agarose clearing screens, which did not detect 3PET‐degrading activity for TA26 (Figs 4 and 5A).

With the diester substrate BHET, TA21 generated both TA and MHET as products, whereas TA26 and TA29 produced only MHET, with small amounts of TA also detected for TA29 (Fig. 5B). When MHET was used as a substrate, only TA21 exhibited significant hydrolytic activity with almost complete substrate conversion to TA, whereas a small amount of TA was detected for TA29 and no MHET cleavage was observed for TA26 (Fig. 5C). Thus, all analysed *T. album* polyesterases demonstrated hydrolytic activity towards 3PET (Fig. 5A) and BHET (Fig. 5B), albeit with differing substrate cleavage preference. Only TA21 exhibited high activity against MHET, resulting in nearly complete conversion to TA, whereas TA29 displayed a clear preference for hydrolysis of terminal ester bonds and produced smaller amounts of TA.

**Fig. 6:**
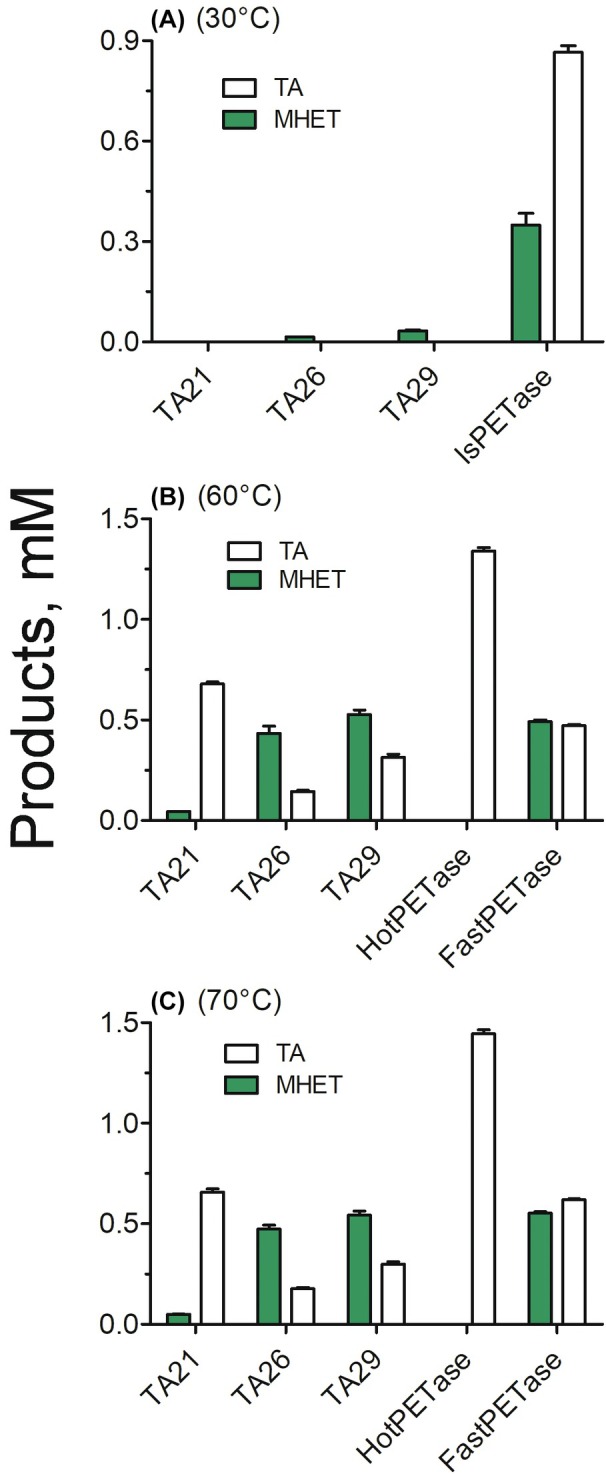
Hydrolysis of amorphous PET by *T. album* polyesterases and benchmark PETases at different temperatures: HPLC analysis of reaction products (MHET and TA). (A), 30 °C; (B), 60 °C; (C), 70 °C. The reaction mixtures (0.2 mL) contained 0.125% of emulsified amorphous PET in 50 mm Tris–HCl buffer (pH 9.0 for TA21 or pH 8.0 for TA26 and TA29) and 30–50 μg of indicated proteins (incubation for 12 h). Results are means ± SEM from at least three independent determinations.

Similar to 3PET, biochemically characterised PETases produce a variable mixture of BHET, MHET, and TA as the main aromatic products of amorphous PET (aPET) degradation [23, 69, 75, 76, 77]. The hydrolytic activity of purified *T. album* polyesterases towards emulsified aPET was evaluated at three temperatures (30 °C, 60 °C and 70 °C) alongside the reference PETases *Is*PETase (at 30 °C), as well as HotPETase and FastPETase (at 60 °C and 70 °C) (Fig. 6). With this substrate, the *T. album* enzymes showed significant degradation of aPET only at 60 °C and 70 °C, producing both MHET and TA as products. This is similar to the archaeal PET‐degrading enzyme PET46, which hydrolysed 3PET at 30 °C, but did not release any aromatic products from semicrystalline PET powder at the same temperature [78]. Like with 3PET, TA21 degraded aPET primarily to TA with small amounts of MHET produced, while MHET was the major product generated by TA26 and TA29 (Fig. 6). Consistent with a previous study [32], *Is*PETase hydrolysed aPET to yield two soluble products, with TA being the predominant product at 30 °C (Fig. 6). HotPETase was the most active aPET‐degrading polyesterase among the tested enzymes, producing TA as the sole soluble product, whereas FastPETase generated similar amounts of TA and MHET at 60 °C and 70 °C (Fig. 6). With aPET as substrate, the three *T. album* polyesterases exhibited hydrolytic activities similar to those of FastPETase and *Is*PETase at 60 °C and 70 °C, although HotPETase was more active than the other enzymes.

**Fig. 7:**
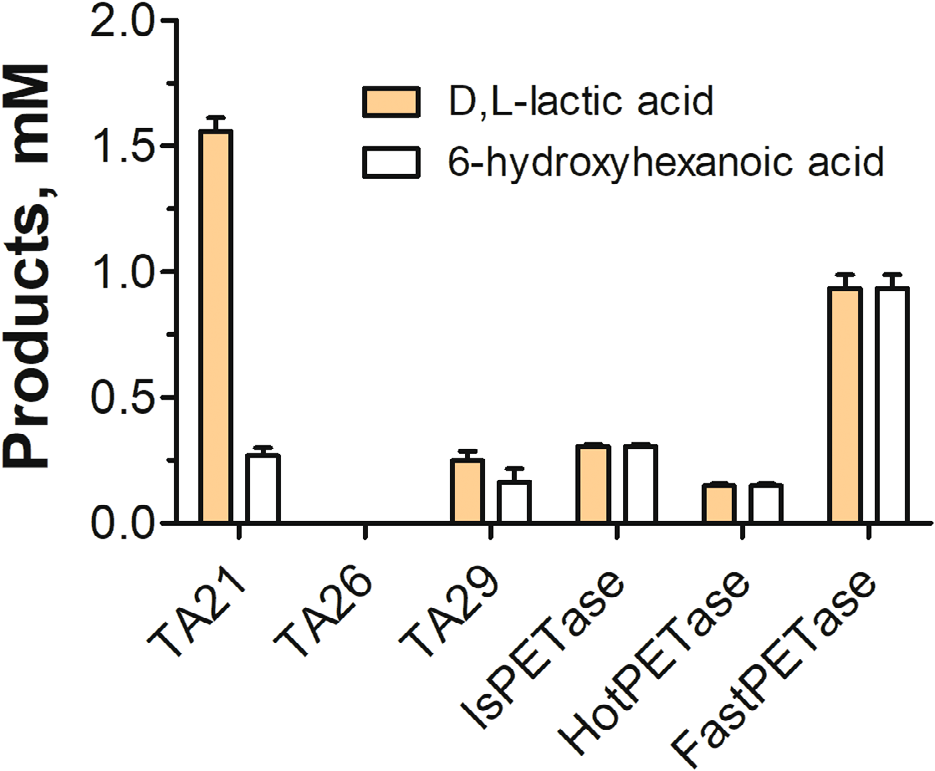
Hydrolysis of PLA and PCL by purified *T. album* polyesterases and benchmark PETases: HPLC analysis of reaction products (d,l‐lactic acid and 6‐hydroxyhexanoic acid). Reaction mixtures (0.2 mL) contained 0.125% of emulsified substrate (PLA or PCL) in 50 mm Tris–HCl buffer (pH 9.0 for TA21, pH 8.0 for TA26 and TA29, pH 9.0 for *Is*PETase, pH 8.0 for FastPETase, and pH 9.2 for HotPETase) and 10 μg of indicated enzymes (incubation at 30 °C for 16 h). Results are means ± SEM from at least three independent determinations.

The hydrolytic activity of the *T. album* polyesterases toward emulsified PLA and PCL (Fig. 4) was further confirmed by HPLC analysis of the soluble reaction products: d,l‐lactic acid (from PLA) and 6‐hydroxyhexanoic acid (from PCL). After overnight incubation at 30 °C, both TA21 and TA29 generated significant amounts of soluble degradation products (d,l‐lactic acid and 6‐hydroxyhexanoic acid) with both polyesters (Fig. 7). In contrast, no detectable PLA or PCL hydrolysis products were observed for TA26 at this temperature, suggesting that higher temperatures are required for PCL degradation by this enzyme (as shown in Fig. 4D). Previously, *Is*PETase was reported to be inactive on aliphatic polyesters (PLA and PBS), as determined by SEM image analysis of plastic films after 96 h of incubation with the enzyme. However, our recent [69] and present (Fig. 7) studies using HPLC analysis of soluble reaction products revealed measurable hydrolytic activity of *Is*PETase and related enzymes (HotPETase and FastPETase) against PLA and PCL at 30 °C, with FastPETase exhibiting the highest activity (Fig. 7). This aligns with recent studies indicating that PET‐degrading enzymes, including the commonly referenced *Is*PETase, LCC, and related proteins, are not strictly specific to PET but can also hydrolyse other polyesters such as PLA and PCL [69, 79, 80]. As shown in Fig. 7, TA21 exhibited the highest PLA‐degrading activity among the enzymes tested under the experimental conditions used.

### Structural analysis of the *T. album* polyesterases

**Fig. 8:**
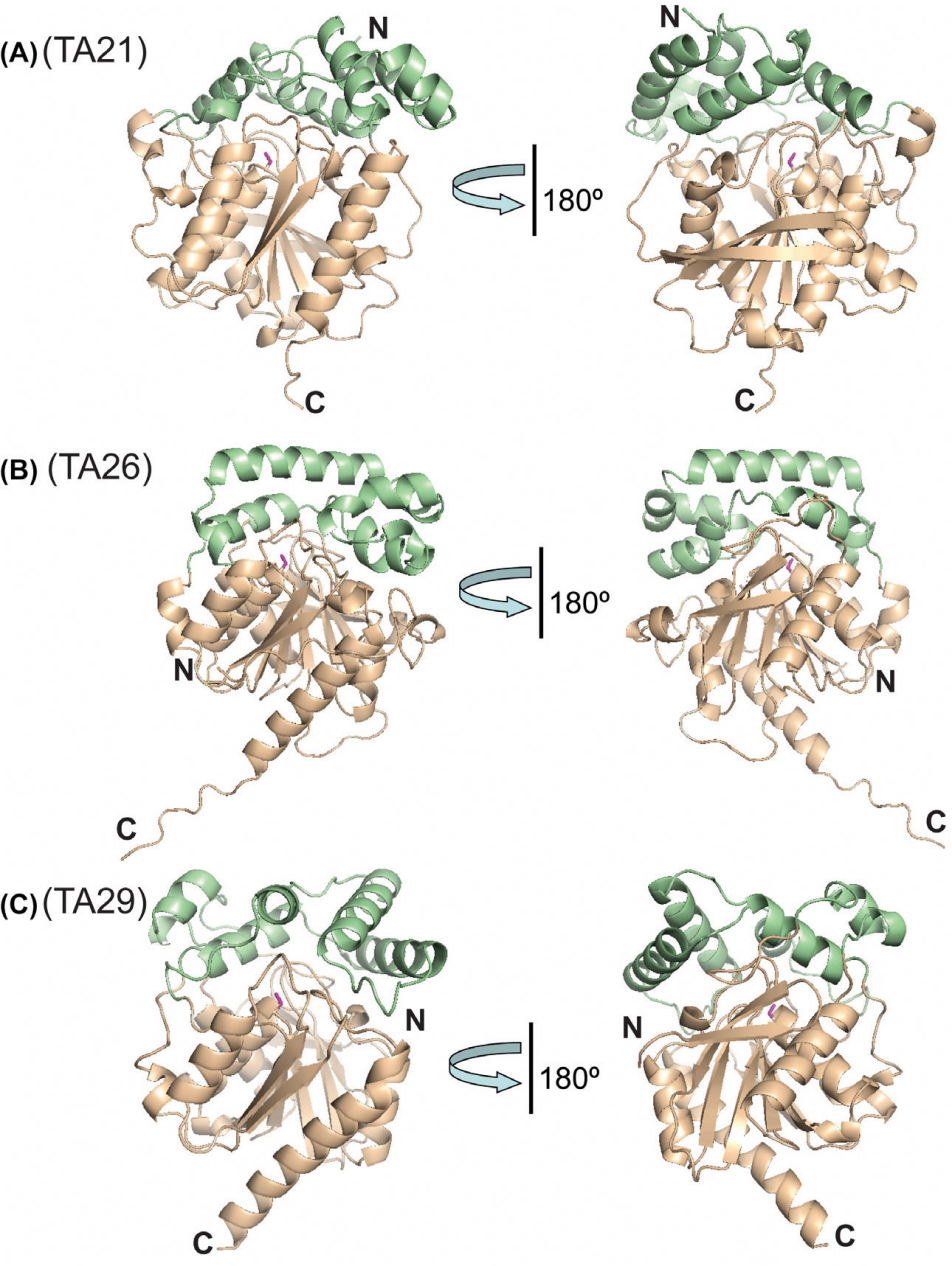
Structural models of *T. album* polyesterases: overall folds and domain organisation. (A) TA21; (B) TA26; (C) TA29. The protein ribbon diagrams present the overall protomer fold, with the core domains coloured wheat and the lid domains coloured light green. Two orientations of each model, related by a 180° rotation, are presented. Active sites are indicated by the side chains of the catalytic serine (magenta‐coloured sticks), whereas protein termini are labelled N and C. Structural models were generated using the alphafold2 server (the ColabFold version) [81].

To gain further insights into the polyesterase activity of *T. album* TA21, TA26 and TA29, high‐quality structural models of these enzymes were generated using the alphafold2 server (the ColabFold version) [81]. The structural models of these proteins revealed two distinct domains: a core domain with a classical α/β hydrolase fold, consisting of a twisted β‐sheet with 8 β‐strands flanked by 5–6 α‐helices, and an α‐helical lid domain composed of 5–6 α‐helices (Fig. 8). Notably, most biochemically characterised PET‐degrading α/β hydrolases, such as *Is*PETase, LCC, PES‐H1 and TfCut2, lack a lid domain [32, 33, 34, 82]. Nevertheless, previous studies on PLA‐ and PET‐degrading enzymes have reported the presence of lid domains in several metagenome‐derived polyesterases, including the archaeal PET46, which hydrolyses PET, BHET, and MHET with activities comparable to or exceeding those of *Is*PETase and LCC [24, 27, 31, 69, 78]. Compared to PET46, the *T. album* polyesterases have substantially larger lid domains between 83 and 107 amino acids (versus 45 aa in PET46) (Fig. 8). The crystal structure of the *I. sakaiensis* MHETase in complex with a nonhydrolyzable MHET analogue (MHETA) revealed a large lid domain (~ 240 aa) composed of nine α‐helices and four β‐strands connected by flexible loops [83, 84]. The structure of the MHETase lid domain highlighted a complex mechanism of MHET recognition, with the lid conferring substrate specificity and contributing several residues essential for MHET binding [83, 84]. Based on the *Is*MHETase structure in complex with MHETA, the core (α/β hydrolase) domain provides the catalytic triad (Ser225, His528, and Asp492), with the substrate ester bond positioned near the side chain of catalytic Ser225, whereas the ester carbonyl oxygen is stabilised by the oxyanion hole formed by the backbone amides of Gly132 and Glu226 [83, 84]. The MHETA phenyl ring forms hydrophobic interactions with the core domain Phe495 and with three aromatic residues in the lid domain (Leu254, Trp397, and Phe415), while Arg411 coordinates the MHETA carboxylate oxygens [83, 84]. Similarly, the metagenomic PETase PET46 exhibit high activity towards MHET, and its active site contains two aromatic residues (Phe39 and Phe148) and Arg119, which are likely involved in MHET binding [78]. Overall, recent experimental and computational studies indicate that substrate recognition by PET‐ and MHET‐hydrolysing enzymes (PETases and MHETases) involves several binding modes and primarily relies on interactions with the MHET phenyl ring and carboxylic group, mediated by hydrophobic (aromatic) and charged/polar (Arg, Gln) residues, respectively [34, 78, 83, 84, 85].

The structural models of the *T. album* polyesterases revealed that their core domains accommodate the catalytic triad (Ser‐His‐Asp) and an oxyanion hole formed by the main‐chain amides of two closely positioned residues (Ala196 and Gly123 in TA21, Met130 and Leu63 in TA26, Met117 and Gly31 in TA29) (Fig. 8). The lid domains of these enzymes cover their active sites in close proximity to the catalytic triads, consistent with a closed conformational state (Fig. 8). Unlike *Is*MHETase, the medium‐sized lid domains of *T. album* polyesterases represent all‐α structures containing multiple hydrophobic and polar/charged residues that may contribute to the binding of various mono‐ and polyester substrates (aPET, 3PET, BHET, MHET) (Fig. 8). Surface charge analysis (electrostatic potential distribution) revealed a high negative charge density around the TA21 active‐site entrance (red regions in Fig. S8), similar to that of *Is*MHETase [84]. In contrast, the corresponding regions in TA26 and TA29 were predominantly positively charged (Fig. S8), and both proteins exhibited a highly polarised overall surface charge distribution, resembling that of *Is*PETase [32]. In all three *T. album* polyesterases, the surfaces opposite the active‐site entrance displayed greater heterogeneity, with distinct clusters of positively and negatively charged residues (Fig. S8). Surface hydrophobicity analysis of the *T. album* polyesterases revealed the predominance of polar residues near the TA21 active‐site entrance, whereas hydrophobic residues were more prevalent in the corresponding regions of TA26 and TA29 (Fig. S9).

**Fig. 9:**
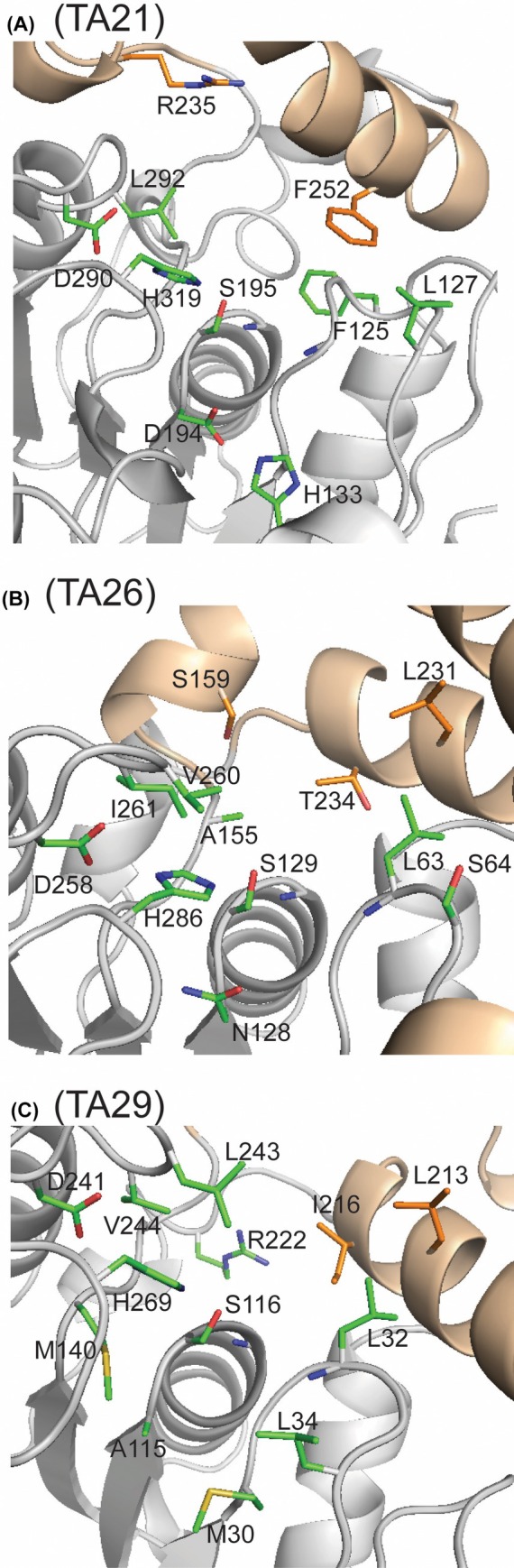
Structural analysis of *T. album* polyesterases: close‐up views of the active sites. (A), TA21; (B), TA26; (C), TA29. Each panel shows amino acid residues potentially involved in MHET binding and the catalytic triads of TA21 (Ser195‐His319‐Asp290), TA26 (Ser129‐His286‐Asp258), and TA29 (Ser116‐His269‐Asp241). Protein ribbon diagrams are coloured grey for the core domain and wheat for the lid domain. Amino acid side chains are shown as sticks, with carbon atoms coloured green in the core domain and orange in the lid domain. Structural models were generated using the alphafold2 server (the ColabFold version) [81].

Structural analysis of the *T. album* polyesterases further indicated that the TA21 binding site for MHET (or an MHET‐based PET unit) includes the catalytic Ser195, as well as the side chains of Phe125, Leu127, and Leu292 in the core domain, and Arg235 and Phe252 in the lid domain (Fig. 9). Similar to the *Is*MHETase‐MHETA complex structure [83], in TA21 the phenyl ring of MHET likely interacts with Phe125, Leu127, Phe252, and Leu292, while the substrate carboxylate group is coordinated by Arg235 (Fig. 9). The side chain of Asp194 is shifted closer (3.1 Å) to His133 and is therefore unlikely to interfere with substrate binding near Ser195. In contrast, the structural models of both TA26 and TA29, which belong to carboxylase family V, revealed only a limited number of aromatic and positively charged residues in their lid domains (Fig. 9). The TA26 active site contained no aromatic residues and Arg for potential interactions with the MHET phenyl ring and carboxylic group, whereas the TA29 model suggested that Arg222 from the core domain may contribute to MHET carboxylate coordination (Fig. 9). Furthermore, the side‐chain carbonyl of Asn128 may interfere with positioning of the cleavable ester group near the catalytic Ser129 in TA26, whereas TA29 contains alanine at this position (Ala115), minimising steric hindrance (Fig. 9). Thus, structural analysis of the active sites of *T. album* polyesterases is consistent with the high hydrolytic activity of TA21 toward MHET, as well as with the moderate activity of TA29 and the low activity of TA26 on this substrate (Fig. 5).

Enzymatic polyester depolymerisation and upcycling have emerged as attractive strategies for advancing plastic recycling within a circular plastic economy [2, 3, 4, 13, 14]. Although several promising PET‐degrading polyesterases have already been identified and engineered, most of them belong to carboxylesterase family V and display high sequence similarity (e.g. LCC, *Is*PETase, PES‐H1) [33, 34, 42, 43, 45]. To unlock the full catalytic potential of nature, future research must expand beyond this single enzyme family and explore other hydrolase families, as well as previously untapped microbial genomes and metagenomes [27, 35, 41, 46]. It is now well‐established that nature employs synergistic combinations of hydrolytic enzymes for the degradation of complex natural polymers, for example endo‐cellulases, exo‐cellulases and beta‐glycosidases for cellulose depolymerisation. Similarly, effective recycling of the growing diversity of synthetic polyesters, particularly heteropolymers, will require rationally designed combinations of complementary enzymes. Enzyme thermotolerance and thermostability remain key prerequisites for efficient polyester depolymerisation and should be prioritised in enzyme discovery and engineering efforts [14, 27, 33, 43]. Furthermore, acid‐ and alcohol‐tolerant enzymes will be important for maintaining catalytic performance in the presence of high concentrations of accumulating reaction products [35, 86]. Continued discovery and biochemical characterisation of novel polyester‐degrading enzymes from extremophilic microorganisms are therefore essential to overcoming current limitations and developing robust enzymatic polyester recycling pipelines. Advancing this research will be central to achieving a sustainable circular plastic economy.

## Materials and methods

### Reagents

Most chemicals and substrates used in this work were of the highest purity (analytical grade) and were purchased from Sigma‐Aldrich/Merck (Gillingham, UK), including *p*‐nitrophenyl (*p*NP) monoesters with varying acyl chain lengths (C2–C14). Similarly, polyester substrates poly(d,l‐lactide) (PLA, molecular weight range M_w_ 10 000–18 000), polycaprolactone (PCL, average M_n_ ~ 10 000) and amorphous PET (aPET, thickness 0.25 mm) were from Sigma‐Aldrich/Merck (Gillingham, UK). The model PET substrate bis(benzoyloxyethyl) terephthalate (3PET, M_w_ 462.4) was produced by CanSyn (Toronto, Canada) using a previously reported protocol [74].

### Gene cloning and recombinant protein purification

Codon‐optimised sequences of the genes of eight selected α/β hydrolases from *T. album* YS‐3 (ATCC 35264) (Tables S1 and S2) were commercially synthesised and cloned into a modified p15TVL vector (Twist Bioscience, CA, USA) for recombinant expression in *E. coli*. Similarly, genes encoding the reference PETase from *I. sakaiensis* (*Is*PETase) and its two engineered variants (HotPETase and FastPETase) were codon‐optimised, synthesised, and cloned into p15TVL using their UniProt entry (A0A0K8P6T7) and published sequences [43, 45]. The plasmids were transformed into *E. coli* LOBSTR cells, which were cultured in 1 L TB medium at 37 °C with shaking at 200 rpm until reaching an optical density (OD_600_) of 0.6–0.8. Protein expression was then induced with 0.4 mm IPTG, and cultures were incubated at 200 rpm for 4 hours followed by overnight at 16 °C. *E. coli* cells were harvested by centrifugation (5000 ***g***) for 20 min at 4 °C and resuspended in binding buffer for affinity protein purification (50 mm HEPES‐K, pH 7.5, 0.4 m NaCl, 5 mm imidazole, 5% v/v glycerol). Cell suspensions were disrupted on ice by sonication at 85 A using a Qsonica Q700 sonicator (Newtown, CT, USA) with 4‐s on/4‐s off pulses for a total of 15 min, and the lysates were clarified by centrifugation at 18 000 ***g*** for 30 min at 4 °C. Recombinant proteins with an N‐terminal 6His‐tag were purified near homogeneity (> 95%) using metal‐chelate affinity chromatography on Ni‐NTA His‐bind resin (Merck, Gillingham, UK). Bound recombinant proteins were washed with washing buffer (50 mm HEPES‐K buffer, pH 7.5, 0.4 m NaCl, 25 mm imidazole, 5% glycerol) and eluted with elution buffer (50 mm HEPES‐K buffer, pH 7.5, 0.4 m NaCl, 250 mm imidazole, 5% glycerol). Protein size and purity were evaluated by SDS/PAGE using 10% Mini‐PROTEAN TGX precast gels (Bio‐Rad, Hercules, CA, USA) with a PageRuler prestained protein ladder (Thermo Fisher Scientific, Basingstoke, UK) as the molecular weight marker (Fig. S10). Following electrophoresis, gels were stained by Ready Blue Protein Gel Stain (Merck, Gillingham, UK). Protein concentrations were determined using the Bradford assay with BSA as the standard.

### Carboxylesterase assays with *p*NP‐monoester substrates

The carboxylesterase activity was measured using the following *p*‐nitrophenyl (*p*NP) ester substrates: *p*NP‐acetate (C2), *p*NP‐butyrate (C4), *p*NP‐hexanoate (C6), *p*NP‐octanoate (C8), *p*NP‐dodecanoate (C12), *p*NP‐tetradecanoate (C14). The stock solutions of *p*NP‐substrates (20 mm) were prepared using acetone as a solvent. The reaction mixtures (0.2 mL, in triplicate) containing 50 mm Tris–HCl buffer (pH 8.0), 1 mm substrate and 1.0 μg purified enzyme were incubated at 30 °C in 96‐well plates. Enzyme activity was monitored spectrophotometrically at 410 nm (millimolar extinction coefficient 17.8 mm ^−1^·cm^−1^) for 20 min, with measurements taken every minute and 5‐s shaking intervals between reads, using a SpectraMax M3 plate‐reader (Molecular Devices, San Jose, CA, USA). To determine the kinetic parameters (*K*_m_ and *k*_cat_) of purified *T. album* α/β hydrolases, carboxylesterase activity was measured against a substrate concentration range of 0.01–2.5 mm at 30 °C. *K*_m_ and *k*_cat_ values were calculated by nonlinear regression of the raw data fitted to the Michaelis–Menten function using GraphPad prism 5.0 (Boston, MA, USA). Enzyme activity was calculated as units (U), where one unit is defined as the amount of enzyme catalysing the conversion of 1 μmol of substrate per minute under the assay conditions described, and specific activity is expressed as U·mg^−1^ of total protein.

### Effects of reaction conditions on carboxylesterase activity and thermostability

The effects of reaction conditions (pH and temperature) and additives (Tween 20 and NaCl) on the carboxylesterase activity of the purified enzymes (TA21, TA26 and TA29) were analysed using a spectrophotometric assay with 1 mm substrates (*p*NP‐hexanoate for TA21 and TA26, *p*NP‐butyrate for TA29). For the analysis of pH effects on enzyme activity, the universal Britton–Robinson buffer system (50 mm acetic acid, 50 mm phosphoric acid, 50 mm boric acid, pH from 3.0 to 11.0) was used as previously described [69, 87]. The effects of temperature, Tween 20, and NaCl on the carboxylesterase activity were characterised at pH 9.0 (for TA21, 50 mm Tris–HCl buffer) or pH 8.0 (for TA26 and TA29, 50 mm Tris–HCl buffer). The reactions were carried out in multiwell plates and monitored at 410 nm using a SpectraMax M3 plate‐reader (Molecular Devices, San Jose, CA, USA). For activity‐based thermostability analyses, the purified proteins (TA21, TA26, and TA29; 1 mg/mL) were preincubated for 1 h at the indicated temperatures (from 20 °C to 90 °C), and residual carboxylesterase activity was then measured spectrophotometrically at 410 nm and 30 °C using 1 mm substrates as indicated above.

### Analysis of protein thermostability using differential scanning fluorimetry (DSF)

Overall thermal stability and melting temperatures (*T*_m_) of purified TA21, TA26 and TA29 were determined using differential scanning fluorimetry (DSF) [88, 89]. Measurements were performed on a QuantStudio 6 Flex Real‐Time PCR system (Thermo Fisher Scientific, Basingstoke, UK) under the following conditions: a temperature range from 25 °C to 95 °C, a heating rate of ~ 1.0 °C·min^−1^, and fluorescence recorded at 1 °C intervals with excitation at 450/490 nm and emission at 560/580 nm. Reaction mixtures (30 μL, prepared in triplicate) contained 10 μg of protein in 50 mm Tris–HCl buffer (for TA21 and TA29: pH 9.0, for TA26: pH 8.0) supplemented with 0.006% Tween 20 and 25x SYPRO Orange® (Invitrogen, Carlsbad, CA, USA). Protein melting temperatures (*T*_m_) were calculated by fitting sigmoidal denaturation curves to a Boltzmann equation using GraphPad prism 5.0 (USA).

### Agarose clearing screens for polyesterase activity with emulsified polyesters

Emulsions of polylactic acid (PLA, poly(d,l‐lactide), M_w_ 10 000–18 000), polycaprolactone (PCL, average M_n_ ~ 10 000), the model PET substrate bis(benzoyloxyethyl) terephthalate (3PET) and amorphous PET (aPET) were prepared in 50 mm Tris–HCl buffer (pH 8.0) as described previously [31]. For agarose‐based polyesterase screens, plastic emulsions were solidified in agarose (2%, w/v), supplemented with kanamycin (50 μg/mL) to prevent microbial growth during incubation. Sample wells (3 mm in diameter) were cut into agarose plates and loaded with 10–50 μg of protein per well. As a negative control, the elution buffer for recombinant protein purification was added to the wells. The plates were sealed with Parafilm and incubated at 37 °C for 2 days, followed by an additional day at 60 °C. The presence of polyesterase activity was indicated by the formation of clear halos around wells with active enzymes.

### Polyesterase assays in solution and HPLC product analysis

To assess polyesterase activity in solution, emulsified polyesters (3PET, aPET, PLA and PCL) were incubated with purified proteins, and the reaction products of enzymatic polyester degradation were analysed by HPLC. Reaction mixtures (200 μL) contained 0.125% of emulsified polyesters in 50 mm Tris–HCl buffer (pH 8.0) and 10 μg of purified enzymes (TA21, TA26, TA29, *Is*PETase, HotPETase, or FastPETase). After overnight incubation at 30 °C with shaking (600 rpm), reaction mixtures were filtered through centrifugal spin filters (10 kDa cut‐off, Amicon, UK), and the resulting flow‐through solutions were used for HPLC analysis of polyester degradation products.

Based on previous research [75, 76], hydrolytic degradation of the model PET substrate bis(benzoyloxyethyl) terephthalate (3PET) is expected to yield a range of possible aromatic products, including bis(2‐hydroxyethyl) terephthalate (BHET), mono(2‐hydroxyethyl) terephthalate (MHET), benzoic acid (BA), hydroxyethyl benzoate (HEB) and terephthalic acid (TA) (Fig. S7). These reaction products were analysed using hydrophobic (reverse phase) chromatography on a C18 column (4.5 × 15 mm, 4.6 μm, Shimadzu) at 40 °C, using a 5–75% methanol gradient mix in 0.1% H_3_PO_4_ acid (detection at 240 nm, flow rate 1 mL/min). Formation of d,l‐lactic acid (from PLA) and 6‐hydroxyhexanoic acid (from PCL) was assessed by ion‐moderated partition chromatography on an Aminex HPX‐87H column (Bio‐Rad, Hercules, CA, USA) at 50 °C with 5 mN isocratic sulfuric acid as the mobile phase (detection at 210 nm, flow rate 0.6 mL/min). All samples were analysed in triplicate; the hydrolytic products were identified by comparison of the retention times with authentic standards, and reactions without enzyme were used as negative controls. Enzymatic hydrolysis of BHET and MHET, two major intermediates of 3PET degradation [75], was examined in reaction mixtures (200 μL) containing 50 mm Tris–HCl buffer (pH 8.0), 5 mm substrate (BHET or MHET, dissolved in DMSO), and 10 μg enzyme (TA21, TA26 or TA29; overnight incubation at 30 °C with shaking at 600 rpm). Formation of reaction products of BHET and MHET hydrolysis (MHET and TA) was evaluated by HPLC on a C18 column as described above.

### Bioinformatics and structural analyses

The presence of signal peptides in the sequences of *T. album* carboxylesterases was analysed by SignalP 6.0 [90]. Coding sequences were partially codon‐optimised using JCat program for CAI 50–60% [91]. Multiple sequence alignments of *T. album* carboxylesterases were constructed using the MAFFT online service [92] and visualised with STRAP [93]. Structural protein models of *T. album* polyesterases were generated using alphafold2 v2.2.4 (DeepMind) [81]. The UCSF chimera x 1.14 software was used for protein structural analysis, including surface charge and hydrophobicity [94]. Enzyme kinetic parameters were determined, and enzyme activity graphs were generated using GraphPad prism software (version 5.00, USA). The protein Neighbour‐joining phylogenetic trees were constructed using protein sequences of *T. album* polyesterases (TA21, TA26, and TA29) and homologous carboxylesterases from PDB database using MUSCLE alignment tool [95] and MEGA11 package [96].

## Conflict of interests

The authors declare no conflicts of interest.

## Author contributions

TNC, ANK, and HM contributed to methodology, investigation, data analysis, writing, and editing of the manuscript. PNG, AFY, MF and OVG conceptualised and designed the research, contributed to data analysis, writing and editing of the manuscript. All authors reviewed and approved the final version of the manuscript.

## Supporting information


**Fig. S1.** Multiple sequence alignment of *T. album* TA21 and related proteins.
**Fig. S2**. Multiple sequence alignment of *T. album* TA26 and TA29 and related proteins.
**Fig. S3**. Phylogenetic analysis of TA21.
**Fig. S4**. Phylogenetic analysis of TA26 and TA29.
**Fig. S5**. Effect of Tween 20 on carboxylesterase activity of purified *T. album* α/β hydrolases.
**Fig. S6**. Thermostability of *T. album* carboxylesterases: differential scanning fluorimetry.
**Fig. S7**. Molecular structures of 3PET and aromatic products of its degradation.
**Fig. S8**. Structural analysis of *T. album* polyesterases: surface electrostatic potential.
**Fig. S9**. Structural analysis of *T. album* polyesterases: surface hydrophobicity.
**Fig. S10**. SDS/PAGE analysis of purified α/β hydrolases from *T. album*.
**Table S1**. List of α/β hydrolases from *T. album* cloned for recombinant expression in *E. coli* and affinity purification in this study.
**Table S2**. Amino acid sequences of eight *T. album* α/β hydrolases cloned in this study.

## Data Availability

The data that support the findings of this study are available in the main text and Supporting Information. Sequences of analysed proteins are presented in the Table S2. Structural models are available in ModelArchive (modelarchive.org) with the accession codes: ma‐ccf3i, ma‐suvzw, ma‐f70rh.
