# Supplementary material for: Polyesterase activity and thermostability of carboxylesterases from Thermoleophilum album YS‐3
Source: FEBS J. 2026 Mar 9;293(15):4724–43. doi: 10.1111/febs.70478 (PMC13440614; doi:10.1111/febs.70478)
Supplement: Supplementary file 1 [file FEBS-293-4724-s001.pdf]

SUPPLEMENTARY MATERIALS

Polyesterase activity and thermostability of carboxylesterases from *Thermoleophilum album* YS-3

Tatyana N. Chernikova, Anna N. Khusnutdinova, Hairong Ma, Manuel Ferrer, Olga V. Golyshina, Alexander F. Yakunin and Peter N. Golyshin

Supplementary Figures

Figure S1. Multiple sequence alignment of *T. album* TA21 and related proteins.

Figure S2. Multiple sequence alignment of *T. album* TA26 and TA29 and related proteins.

Figure S3. Phylogenetic analysis of TA21.

Figure S4. Phylogenetic analysis of TA26 and TA29.

Figure S5. Effect of Tween 20 on carboxylesterase activity of purified *T. album* α/β hydrolases.

Figure S6. Thermostability of *T. album* carboxylesterases: differential scanning fluorimetry.

Figure S7. Molecular structures of 3PET and aromatic products of its degradation.

Figure S8. Structural analysis of *T. album* polyesterases: surface electrostatic potential.

Figure S9. Structural analysis of *T. album* polyesterases: surface hydrophobicity.

Figure S10. SDS-PAGE analysis of purified α/β hydrolases from *T. album*.

Supplementary Tables

Table S1. List of α/β hydrolases from *T. album* cloned for recombinant expression in *E. coli* and affinity purification in this study.

Table S2. Amino acid sequences of eight *T. album* α/β hydrolases cloned in this study.

```
EstD11    1 MA                                      SEALTMIVNLLRSQ    RPLQEPTVEQMRAGL    31
IS12      1 MA                                     LDPGAAALLQQMAQ  AGVPPFNQMTVQQARQSM    34
PestE     1 MP                                     LSPILRQILQQLAAQLQFRPDMDVKTVREQFEKS    36
TA21      1 VPPADLQARLAKPLVRSLGRLPRPALAKLAGPPPAAAPDLEPEAQLLARFSER  SPLPRIETLPVAAARSLF    71

EstD11   32 EAMAQMSP    LPAD VELTTVDAGGVP GAWVRVPESDPDR  VVLYLHGGGYVIGSIRTHRDLAQRIARA    96
IS12     35 TGLAGTAPK   YPVAKVEDRTIPGPAGQIPVRIYWPISGQKV GLVVYYHGGGWVICDLETHDGTCRQLANA   103
PestE    37 SLILVKMAN   EPIHRVEDITIPGRGGPIRARVYRPRDGERL PAVVYYHGGGFVLGSVETHDHVCRRLANL   105
TA21     72 DAQVDSVTRREHLPVTTLE HTLPVGDVRLRARLYVPQQAPSTGPLLVYFHGGGFVLGSIATHDAPLRLLAHA   143

EstD11   97 ARCRVLLIDYRLAPEHPHPAAVEDSTRAYRWLLET    GSDPKRMAIAGDSAGGGLTVATLVALRDAGVPLP   165
IS12    104 SDCIVMSVDYRLAPEHKFPAAVEDCYAAAAWAAENAATFGVDPSRLVVAGDSAGGNLAAVVALLAKERGGPRL   176
PestE   106 SGAVVVSVDYRLAPEHKFPAAVEDAYDAAKWVADNYDKLGVDNGKIAVAGDSAGGNLAAVTAIMARDRGESFV   178
TA21    144 SGVRILSVDYRLAPEHRFPAAVEDAVAAFEHVAAEPQLFSTDPGSIAVGGDSAGGTLAAVVCQELRRRGGPLP   216

EstD11  166 AAAVCLSPWVDLEGIGESMTTKAAVD  PMVQREPLLRMASMYL AGQDPRTPLAAPLYA DLRGLPPLLIQV   234
IS12    177 AHQVLIYPVTDYSFDRPSYRENAEG   YLLTRDAMEWFWRHYLNSDADGQNPLASPLRAPDLRGLPSATIIT   246
PestE   179 KYQVLIYPAVNLTGSPTVSRVEYSGPEYVILTADLMAWFGRQYFSKPQDALSPYASPIFA DLSNLPPALVIT   250
TA21    217 CFQLLIYPATELFDQFPSRRAFARG   FILTEEEMNWFADHYVPDRELRRDPRVSPLREPDLAGLPPAHVAT   286

EstD11  235 GTAETLLDDSVRLAERARAAGVQVTLEPWEDMIHVWQAFAAMLPEGQQAIERIGEFLRQHWQ             296
IS12    247 AEYDPLRDEGEAYAQRLMEAGVPTTCVRYLGQIHGFVTLEHVLPAGRQALLALGATIKGVLTGALGGQPAAA   318
PestE   251 AEYDPLRDEGELYAHLLKTRGVRAVAVRYNGVIHGFVNFYPILEEGREAVSQIAASIKSMAV         A   313
TA21    287 AAADPLRDEGEEYARRLRAAGVPVSAYRHP HLHGFLNMT ALPAARAGTIQIAGVLCHALS    AVPREP   352
```

Figure S1. Multiple sequence alignment of TA21 and biochemically characterised Family IV carboxylesterases: EstD11(UniProt ID A0A8I3AZT7), IS12 (UniProt ID A0A7C2Y9X0), and PestE (UniProt ID Q8NKS0). Catalytic triad residues (Ser, His, Asp) are shown in red, while other conserved residues are highlighted in bold. Multiple sequence alignments of *T. album* carboxylesterases were constructed using the MAFFT online service [92] and visualised with STRAP [93].

```
ISPETase   1 QTNP YARGPNPTAASLEAS                          AGPFTVRSFTVSR  PSGYGAGTVY      42
LCC        1 MSNP YQRGPNPTRSALTAD                           GPFSVATYTVSRLSVSGFGGGVIY      43
TA26       1 VSDGGVVRGPDDY  GLTAEPNWRTVDWARHLARVRIGRTPVNYVRLDPPQRRDGAVPVVFVHGLSGQWQN      69
TA29       1 VAES FAK                            IGSIELCYEDLGPADGE    PLLLVMGLGLQLVH      58

ISPETase  43 YPTNAGGTVGAIAIVPGYTARQSSIKWWGPR LASHGFVVITIDTNSTLDQPSSRSSQQMAALRQVASLNG     112
LCC       44 YPTGTSLTFGGIAMSPGYTADASSLAWLGRR LASHGFVVLVINTNSRFDYPDSRASQLSAALNY   LRT     110
TA26      70 WLEN    LPATALER  PAVALDLPGFGRSPLPANGTVSI          P                YYA     108
TA29      39 WEDG    FCELLRERGFRVVRFDNRDAGRSSKIKGG              PRPNIARALVGDRRVAAYSL      91

ISPETase 113 TSSSP    IYGKVDTARMGVMGWSMGGGGSLISAANNP                                     147
LCC      111 SSPSA    VRARLDANRLAVAGHSMGGGGTLRIAEQNP                                     145
TA26     109 SVVAR    LCAALGFDRYVVCGNSMGGLIAAELALREPGRVQGLV LVSPAGVSSATVKVWPAVAVGTVV     174
TA29      92 SDMARDAVGLLDHLGIERAHVVGASMGGMIGQLMAIEHPQRVASLCSIMSSAG                       144

ISPETase 148                              SLKAAAPQA                     PWDSST          162
LCC      146                              SLKAAVPLT                     PWHTDK          160
TA26     175 RLVGVYGGRFHRHVARRR    RARALGLALVARYPQRLDADLA CEGLIKGAGKPGFAPALRATV      235
TA29     145                 RRTDLPRLRALG ALLARPPRERDRFVAHMERIFRVIGSPAY PTPRERLRELAA     197

ISPETase 163                             NFSSVTVPTLIFACENDSIAPVNSSALPIYDSM SRNAKQFLE     204
LCC      161                             TFNT SVPVLIVGAEADTVAPVSQHAIPFYQNLPSTTPKVYVE     202
TA26     236  GYD                     LRERLPEIAVPTLVVWGKDDRVIPVRDADVWAS    RIANTEKVI     280
TA29     198 IAYDRGHSTAGTARQLVAILTAPSRVR RLRAVQVPTLVVHGGADPLVPPRAGRAVAV    AVPGARYVE     263

ISPETase 205 INGGSHSC ANSGNSNQALIGKKGVAWMKRFMDNDTRYSTFACENPNSTRVSDFRTAN  CS           264
LCC      203 LDNASHF    APNSNNAAISVYTISWMKLWVDNDTRYRQFLC NVNDPALSDFRTNNRHC          258
TA26     281 LPRTGHVAQLERPRTFN           RLLHDFVARV      DAQEHTPTTAASAS             321
TA29     264 FAGMGH    DLPR              ELWPEIVDHI      AANTRRATASAAA              296
```

Figure S2. Multiple sequence alignment of *T. album* TA26 and TA29 with biochemically characterised polyesterases belonging to the Family V carboxylesterases: *Is*PETase from *I. sakaiensis* (UniProt ID A0A0K8P6T7) and LCC from a leaf-branch compost metagenome (UniProt ID G9BY57). Catalytic triad residues (Ser, His, Asp) are shown in red, while other conserved residues are highlighted in bold. Multiple sequence alignments of *T. album* carboxylesterases were constructed using the MAFFT online service [92] and visualised with STRAP [93].

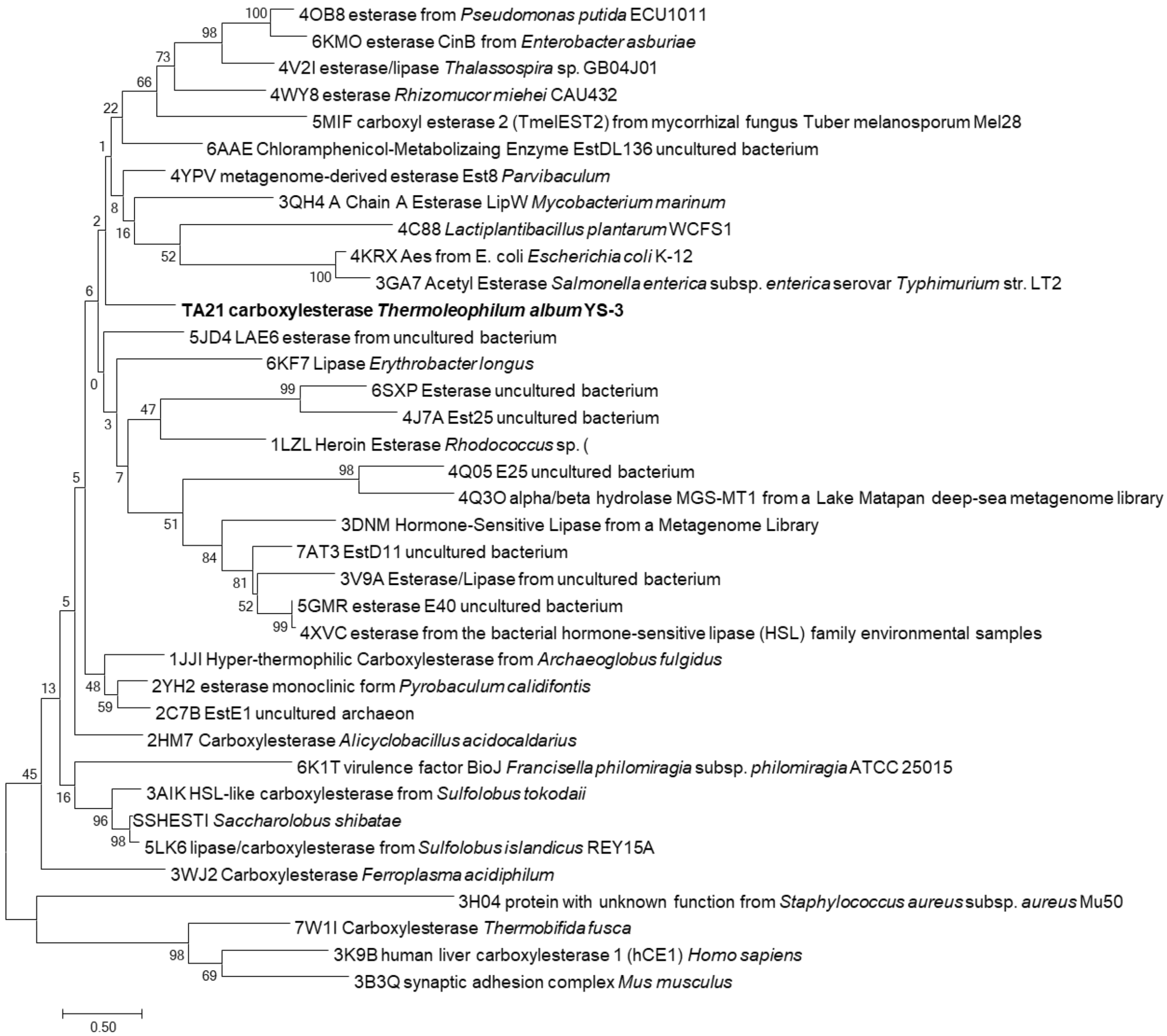

Figure S3. Phylogenetic analysis of TA21: a Neighbour-Joining phylogenetic tree of TA21 and homologous proteins from PDB. The tree was constructed in MEGA11 package [96] based on Muscle alignment tool (Kimura model) [95], with 1,000 pseudo replicates used for bootstrap analysis . Scale bar, 0.5 substitutions per position.

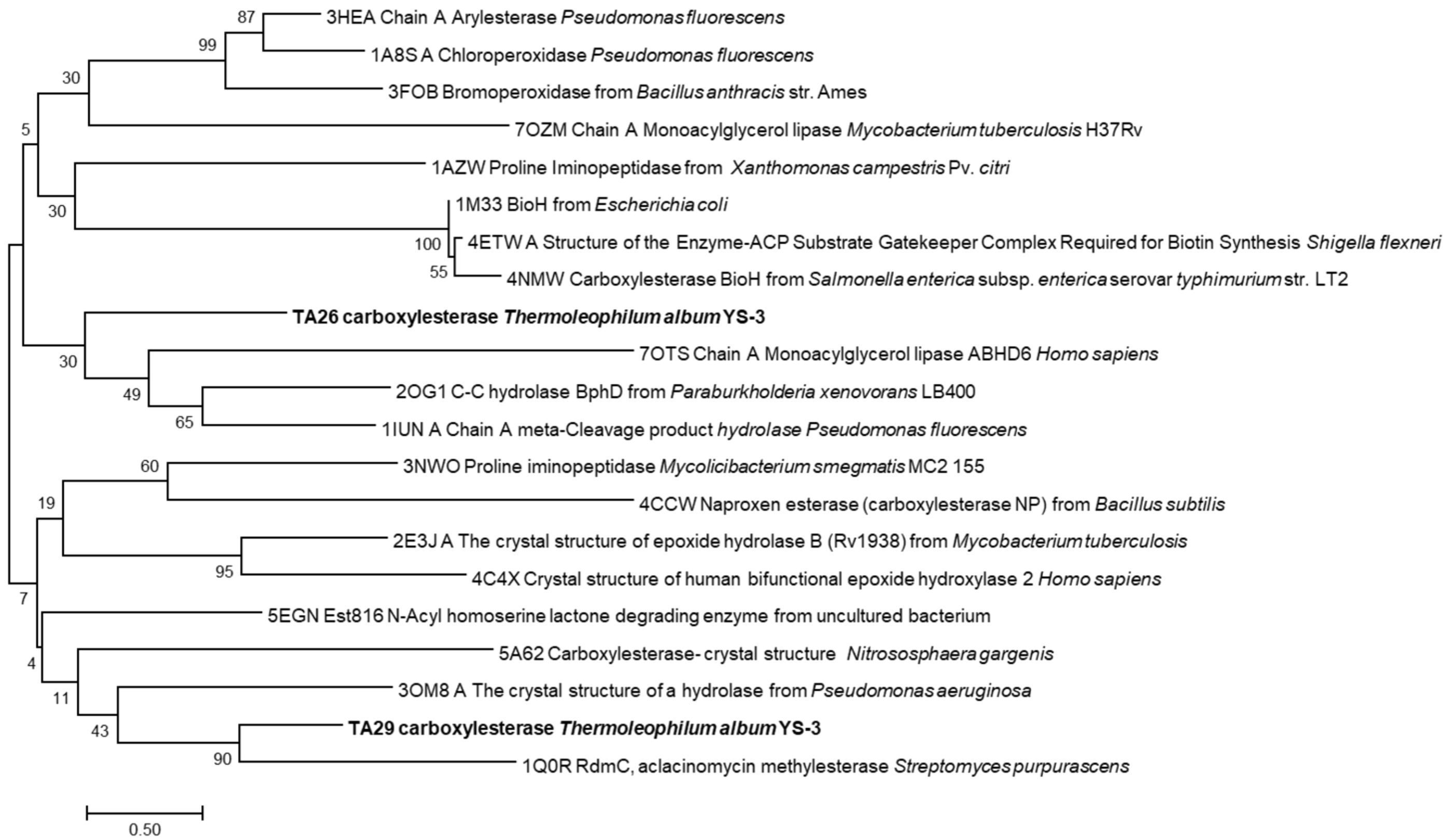

Figure S4. Phylogenetic analysis of TA26 and TA29: a Neighbour-Joining phylogenetic tree of TA26 and TA29 and homologous proteins from PDB. The tree was constructed in MEGA11 package [96] based on Muscle alignment tool (Kimura model) [95], with 1,000 pseudo replicates used for bootstrap analysis. Scale bar, 0.5 substitutions per position.

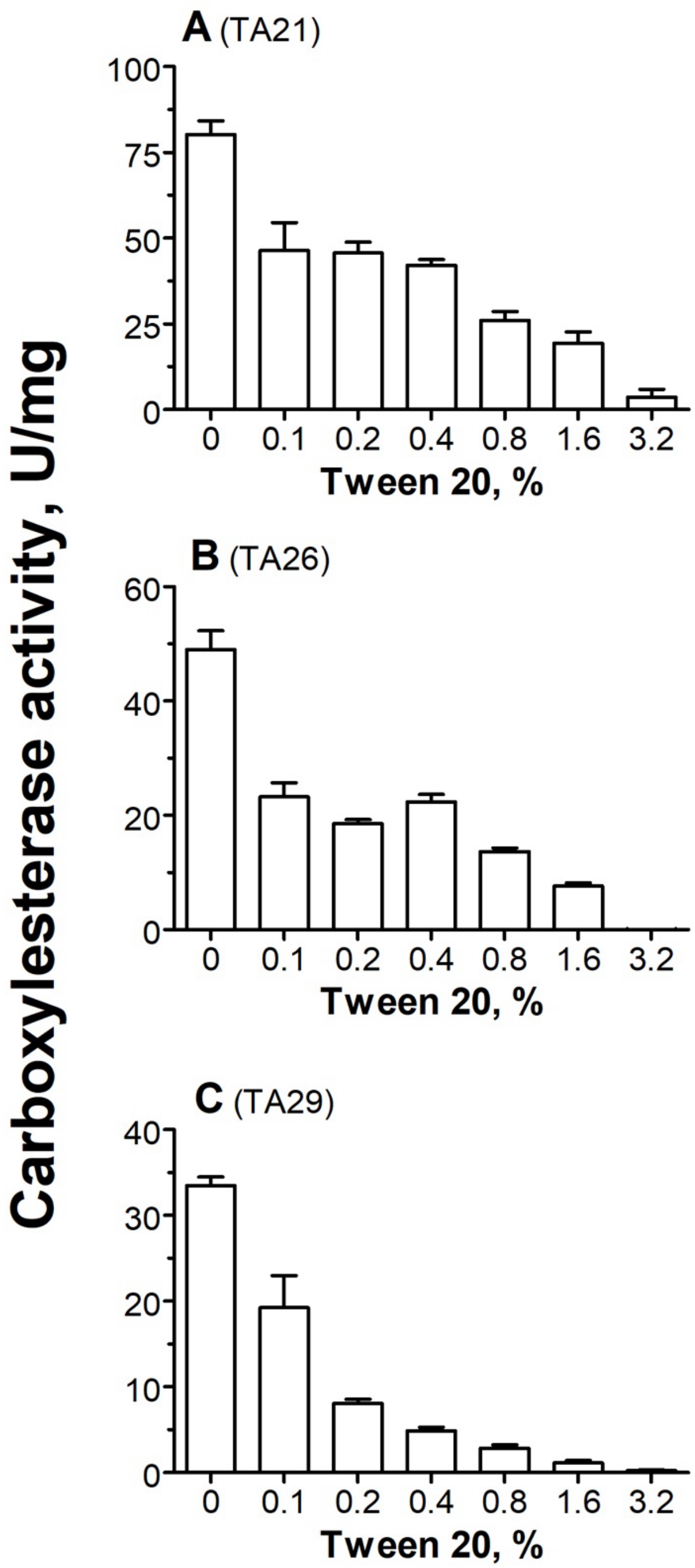

Figure S5. Effect of Tween 20 on carboxylesterase activity of *T. album* carboxylesterases: (**A**), TA21; (**B**), TA26; (**C**), TA29. The reaction mixture (0.2 ml) contained 50 mM Tris-HCl buffer (pH 9.0 for TA21 and pH 8.0 for TA26 and TA29), 1 mM substrate (*p*NP-hexanoate for TA21 and TA26, *p*NP-butyrate for TA29), Tween 20 (as indicated), and 1 µg enzyme (20 min incubation at 30 ºC). Data represent means ± SEM from at least three independent determinations.

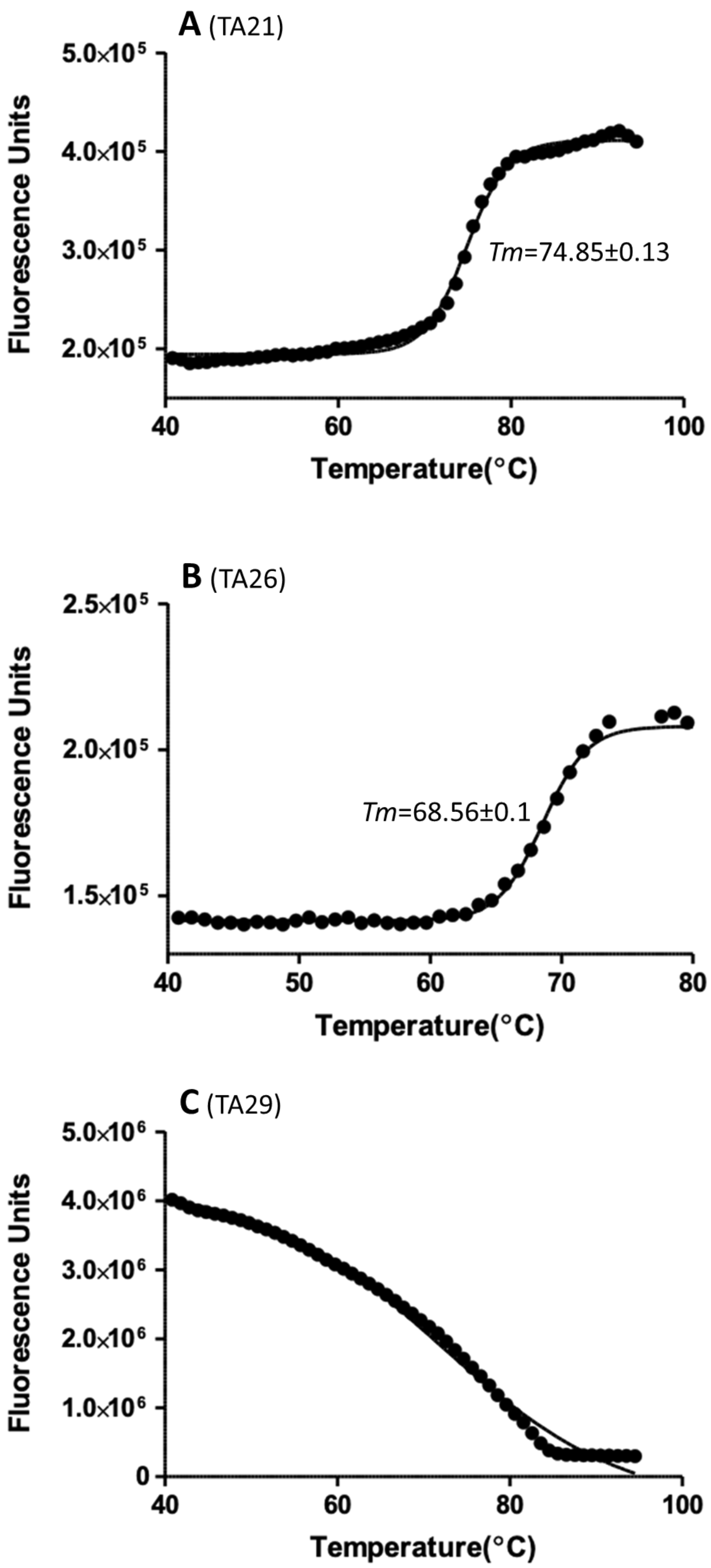

Figure S6. Thermostability of *T. album* carboxylesterases: differential scanning fluorescence. Determination of protein melting points (*Tm*) of TA21(**A**), TA26 (**B**), and TA29 (**C**) using differential scanning fluorescence (DSF) with SYPRO Orange dye. Temperature-induced protein unfolding was monitored in real-time using 10 μg of protein/sample (0.2 ml) in 50 mM Tris-HCl buffer (pH 9.0 for TA21 and pH 8.0 for TA26 and TA29). The observed fluorescence values were fitted to a sigmoidal Boltzmann curve.

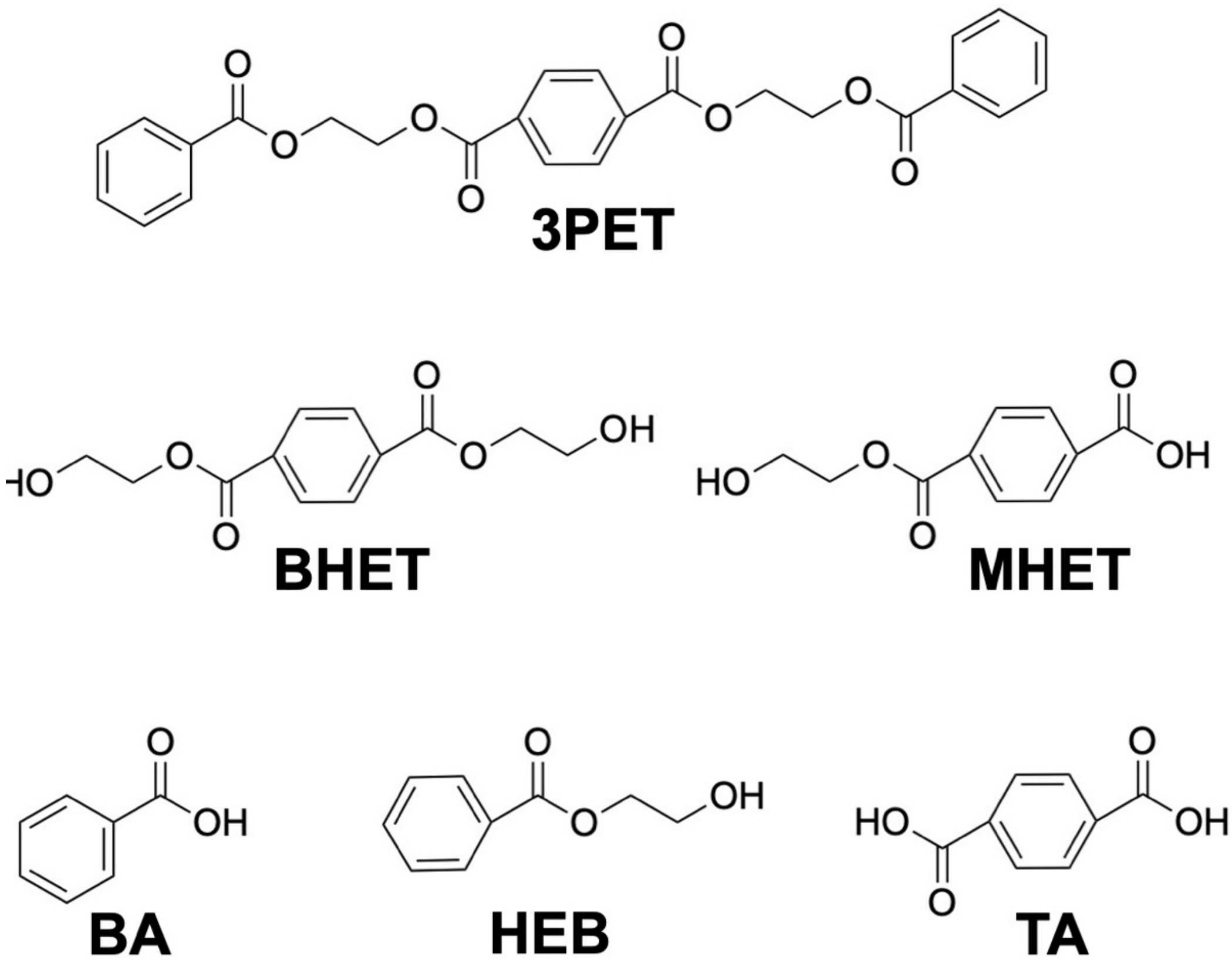

Figure S7. Molecular structures of 3PET and aromatic products of its degradation. 3PET, bis(benzoyloxyethyl) terephthalate; BHET, bis(2-hydroxyethyl) terephthalate; MHET, mono(2-hydroxyethyl) terephthalate; BA, benzoic acid; HEB, hydroxyethylbenzoate; TA, terephthalic acid.

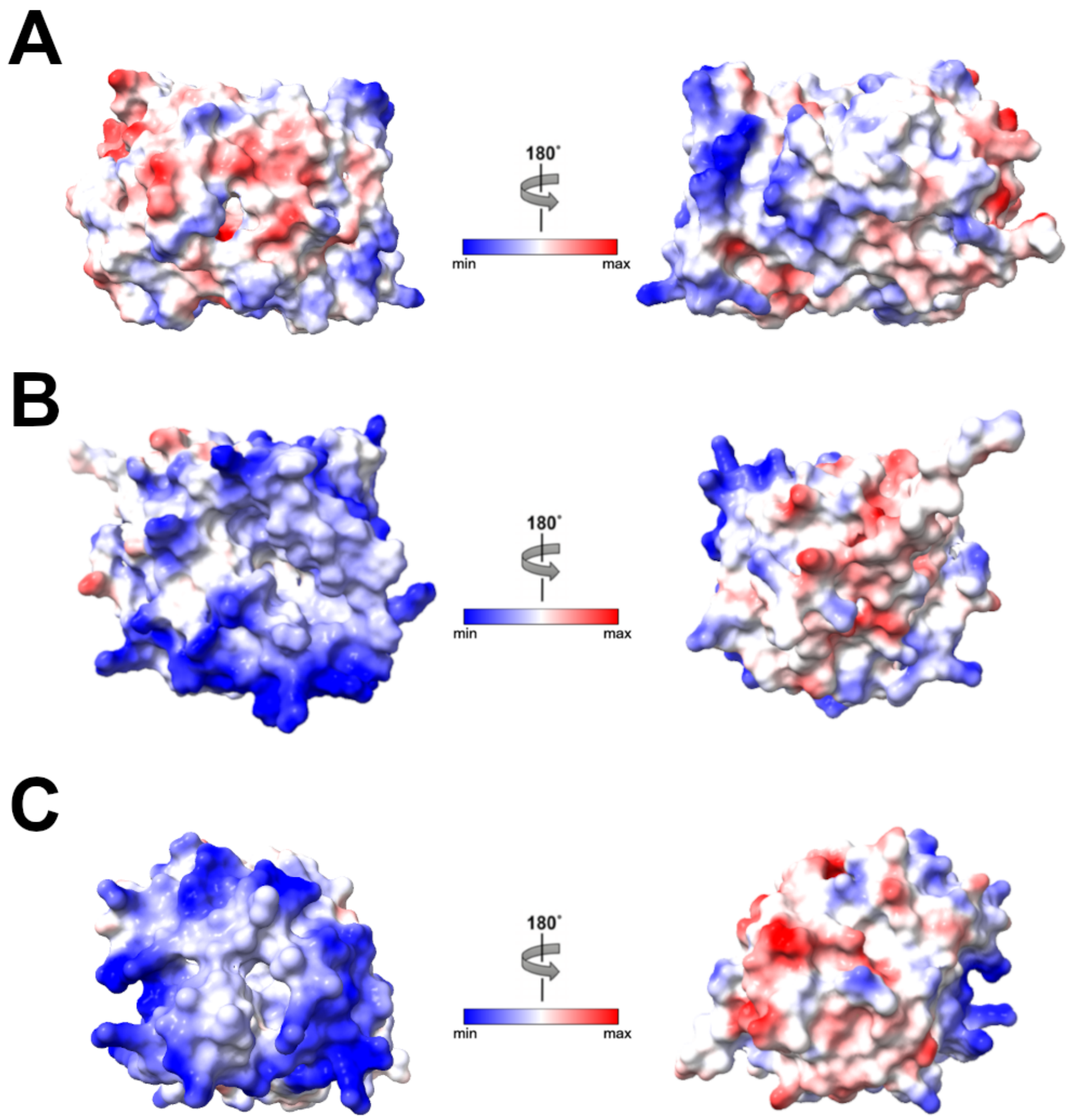

Figure S8. Structural analysis of *T. album* polyesterases: surface electrostatic potential (Coulombic charge) of the protomers. Surface representations of TA21 (**A**), TA26 (**B**), and TA29 (**C**) showing the electrostatic potential of solvent-accessible residues. The distribution of surface potential is shown as a gradient from blue (negatively charged) to red (positively charged), with two views related by a 180° rotation and scale bars indicating the color schemes of the surface-exposed residues. Structural models were generated using the AlphaFold2 server (the ColabFold version) [81]. The UCSF Chimera X 1.14 software was used for protein surface charge analysis [94]

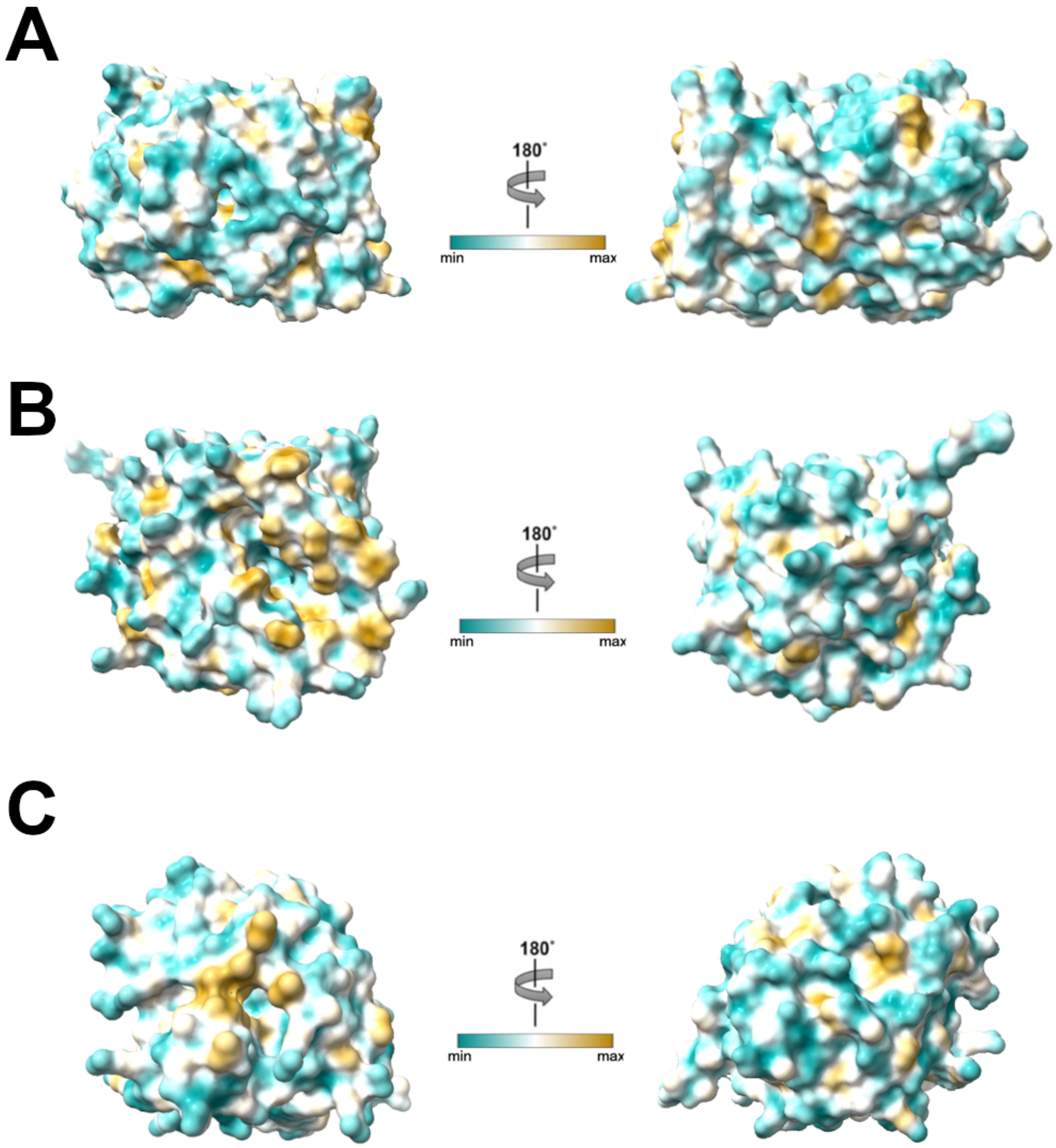

Figure S9. Structural analysis of *T. album* polyesterases: surface hydrophobicity distribution of the protomers. Surface presentations of TA21 (**A**), TA26 (**B**), and TA29 (**C**) showing the hydrophobicity of solvent-accessible residues. The distribution of surface hydrophobicity is presented as a gradient from cyan (polar) to yellow (hydrophobic), with two orientations related by 180° rotation and scale bars indicating the color schemes of the surface-exposed residues. Structural models were generated using the AlphaFold2 server (the ColabFold version) [81]. The UCSF Chimera X 1.14 software was used for protein hydrophobicity analysis [94].

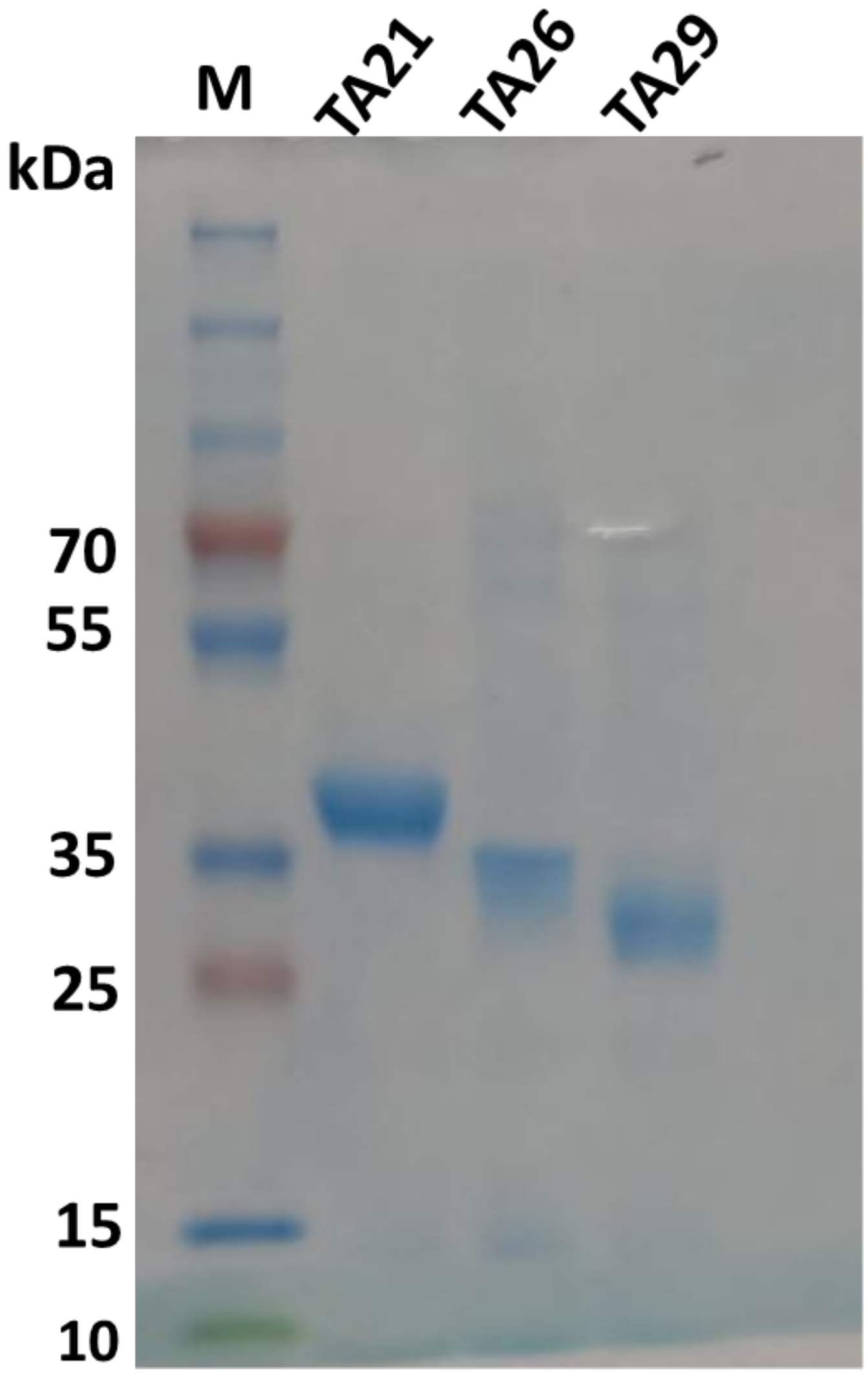

Figure S10. SDS-PAGE analysis of purified α/β hydrolases from *T. album* used in this study. The 6xHis-tagged proteins were recombinantly expressed in *E. coli* cells and affinity purified using nickel-chelate affinity chromatography. Purified proteins (3 µg/well) were resolved on a 10% SDS-PAGE gel and stained with Coomassie Brilliant blue. Lane M: molecular weight markers (kDa), represented by a pre-stained protein ladder.

Table S1. List of α/β hydrolases from *T. album* cloned for recombinant expression in *E. coli* and affinity purification.

Protein name (this study)	Locus tag	GenBank accession #	Protein length (aa)	Signal peptides	Esterase family[a]	Expression & solubility
1. TA3	JDY09_07310	WDT94665.1	319	yes	I	no
2. TA4	JDY09_02990	WDT94235.1	430	yes	II	no
3. TA8	JDY09_02965	WDT94231.1	448	yes	I	no
4. TA9	JDY09_04675	WDT94547.1	591	yes	V	no
5. TA20	JDY09_02620	WDT94165.1	297	no	V	no
6. TA21	JDY09_02080	WDT94064.1	352	no	IV	yes
7. TA26	JDY09_04100	WDT94443.1	321	no	V	yes
8. TA29	JDY09_02750	WDT94189.1	296	no	V	yes

[a] Carboxylesterase family according to Arpigny and Jaeger (1999).

Table S2. Amino acid sequences of eight *T. album* α/β hydrolases cloned in this study.

1. TA3

```
MRMSRRAKLRCALPLALVGVVAAFAPAANAQLAVDYSLPKALAAWAANPNGSPPGANDWSCRPSSLHPYP
VILVHGTFENQRFNWNALAPLLKNAGYCVFSFNYGATQGTQLTGGFANGLAAVRDSARQLAAFVDRVRAA
TGAAEVDIVGHSQGGMMPRYYTNKLGGASKVRALVGLSPSNHGTTLSGLTEFARLLGPIGNAAVQLVTSF
CPACGDQIRGSAFMQELNAGGDTVAGVAYTVIQTKYDEVVTPYDSAFLTPAASSHQVRNILVQNGCPLDL
SEHLAIPFDRRALAYVLNALDPARPRPVPCVPVAPLIGG
```

2. TA4

```
MRGRGAFPALRALALAVLAVFALGAATPNALARARYVALGDSYSSGTGTGNYFDRQCLRSEQAYPAVVTI
ARGDLDLVFEACSGATTDDLLREQLDGLTADTRWVTVTIGGNDIGFADIVVACASASLAGNCEEEIAEGE
RKAREELPAKLDRTYRAIRQRAPHARVLVLGYPRLFQDRGRPPNCTGGALFTPERIERLNRGAEMLRDLI
RERVAAAGPGFYFLDAISVFEGHEVCSPNPWINGVMTEIVSSYHPNRFGHFAGYGRMVLDAMERIPRAER
QSPTSGSRTGTPRSGTGTPNGGSEVRALRRCPVAPRARFTRSGVGRFRLGTSAGSLLRQAGRPQTRSRNA
WTWCVRGGGAVTVVFSRGRTVAVFTNARGHTYRGLGAGDRVTAALRRRLALARRGRYLVARGGVFLRIRQ
GRVVHIGVRS
```

3. TA8

```
MARAQGLALAFTVAIALAMALAVAPGAQAARTALIERYVAIAGAPAPGAARYDRVFAYQVGARRARTVVV
LVPGFLGGAGTIAPVARDIAARLPATQVWVVDRREQALEDQRGFARRSASAAYDYYFGGKYRRVRGLDVP
YASEWGLAVQLADLRRVVLAARKGGRRVVLGGHSLGASTALAYAAWDFAGRPGYRDISGLVLIDGGLLGT
FASADAARARRELDEMRRGQVFEDVLGLGVPELAGIFVQTAGLYALRAPDVPAKLQPLVPAAFRPAFTVT
NEALVGYAFDRDTSPPGFEFVRVRAGRLAAAGDPRPWVSGERTPIERLVRAMAATKPDFIEWYFPRRLRL
DVDAANDLRMTAAAKILGLRLRWARRVDVPLYAYQTDLTGGRVLRGARRFVAISRVRRYRFVDDPAASHL
DPVVGDPRSNRFLKTVVPFLRELERTRR
```

4. TA9

MPLAVRSLRAALALGSVLALLGVAHADAVAAPRPFGHDCTPLSGVRFCPTRDDRERVPSFDGVPLDVDVT
LPPSGDGPFPTIVMLHGWGGSKAMFESASANSAYSNVALASRGYAVVNYSARGWGRSCGVPASRTAGCER
GWTHLADQRYEIRDTQYLLGLLVDQGIADPRALGATGVSYGGIQSLELAFLRDRVRLRNGKLVRWRSPKG
RLLTLRAAWARWGTADLAGSLTLNGRISDARRARVGASWEPLGIPKQSYINGLYSLAAISAYLAPPGADA
TADLTTWKAITDAARETPAARSLVRELTRYHGAAGVADRRRAVAPLLIQNGWTDDLFPAIDGVYAYRLAG
SGRRGFVALQIGDLGHPRAQTRLGEEGVFLRQGLAFFDRFLRGRRGGPGNGAVTAFLQSCPESTPSRPLR
ARSWDSLARGRLILASRASGTVTSDGGDPTVGRTIDPIGGGGACAAVTPTKARGTLVLERRVRRTTVVAG
LPIISMRVRASAGRAYVVARLWSVGRDGKMRLIGRGAVRLRAKQRGAVRFQLLGTGWRLAAGDRVRLELL
GRDAPYLRPNDEEGFKVRVSRVRALLPVVGR

5. TA20

MIALAVAAFEQAATAATRPTATARATAATRTTAASPRPRSIREVPIRTVRAGDGRIGYRSFGRGRPLVLI
MGLGGTMDAWAPSVVDALARHRRVITFDNLGIRRTTLKGPITIPRMARTVASLVRALRLRRADVLGWSMG
GMIAQSLARLYPQRVRRLVLCATAPGDGKATPPKPGTQFGTALFPPNRGDLMAAWSREITSYPNAQPGAP
ANIFQQQLAAIARWIAGRERSGRELRKIRLPVLVAHGALDRLLPVANARHIAHTIRGARLVVYRDAAHNF
LFQHRRDFVARVNAFLR

6. TA21

MPPADLQARLAKPLVRSLGRLPRPALAKLAGPPPAAAPDLEPEAQLLARFSERSPLPRIETLPVAAARSL
FDAQVDSVTRREHLPVTTLEHTLPVGDVRLRARLYVPQQAPSTGPLLVYFHGGGFVLGSIATHDAPLRLL
AHASGVRILSVDYRLAPEHRFPAAVEDAVAAFEHVAAEPQLFSTDPGSIAVGGDSAGGTLAAVVCQELRR
RGGPLPCFQLLIYPATELFDQFPSRRAFARGFILTEEEMNWFADHYVPDRELRRDPRVSPLREPDLAGLP
PAHVATAAADPLRDEGEEYARRLRAAGVPVSAYRHPHLHGFLNMTALPAARAGTIQIAGVLCHALSAVPR
EP

7. TA26

MSDGGVVRGPDDYGLTAEPNWRTVDWARHLARVRIGRTPVNYVRLDPPQRRDGAVPVVFVHGLSGQWQNW
LENLPATALERPAVALDLPGFGRSPLPANGTVSIPYYASVVARLCAALGFDRYVVCGNSMGGLIAAELAL
REPGRVQGLVLVSPAGVSSATVKVWPAVAVGTVVRLVGVYGGRFHRHVARRRRARALGLALVARYPQRLD
ADLACEGLIKGAGKPGFAPALRATVGYDLRERLPEIAVPTLVVWGKDDRVIPVRDADVWASRIANTEKVI
LPRTGHVAQLERPRTFNRLLHDFVARVDAQEHTPTTAASAS

8. TA29

MAESFAKIGSIELCYEDLGPADGEPLLLVMGLGLQLVHWEDGFCELLRERGFRVVRFDNRDAGRSSKIKG
GPRPNIARALVGDRRVAAYSLSDMARDAVGLLDHLGIERAHVVGASMGGMIGQLMAIEHPQRVASLCSIM
SSAGRRTDLPRLRALGALLARPPRERDRFVAHMERIFRVIGSPAYPTPRERLRELAAIAYDRGHSTAGTA
RQLVAILTAPSRVRRLRAVQVPTLVVHGGADPLVPPRAGRAVAVAVPGARYVEFAGMGHDLPRELWPEIV
DHIAANTRRATASAAA
